# Maize transformation technology development for commercial event generation

**DOI:** 10.3389/fpls.2014.00379

**Published:** 2014-08-05

**Authors:** Qiudeng Que, Sivamani Elumalai, Xianggan Li, Heng Zhong, Samson Nalapalli, Michael Schweiner, Xiaoyin Fei, Michael Nuccio, Timothy Kelliher, Weining Gu, Zhongying Chen, Mary-Dell M. Chilton

**Affiliations:** Syngenta Biotechnology, Inc.Research Triangle Park, NC, USA

**Keywords:** maize transformation, *Agrobacterium*, T-DNA, transgenic events, multi-gene stack, trait development, genome modification, targeted insertion

## Abstract

Maize is an important food and feed crop in many countries. It is also one of the most important target crops for the application of biotechnology. Currently, there are more biotech traits available on the market in maize than in any other crop. Generation of transgenic events is a crucial step in the development of biotech traits. For commercial applications, a high throughput transformation system producing a large number of high quality events in an elite genetic background is highly desirable. There has been tremendous progress in *Agrobacterium*-mediated maize transformation since the publication of the Ishida et al. (1996) paper and the technology has been widely adopted for transgenic event production by many labs around the world. We will review general efforts in establishing efficient maize transformation technologies useful for transgenic event production in trait research and development. The review will also discuss transformation systems used for generating commercial maize trait events currently on the market. As the number of traits is increasing steadily and two or more modes of action are used to control key pests, new tools are needed to efficiently transform vectors containing multiple trait genes. We will review general guidelines for assembling binary vectors for commercial transformation. Approaches to increase transformation efficiency and gene expression of large gene stack vectors will be discussed. Finally, recent studies of targeted genome modification and transgene insertion using different site-directed nuclease technologies will be reviewed.

## Introduction

Maize is an important food and feed crop around the world. Since the market launch of the first transgenic Bt maize products in the mid-1990s, maize has become one of the most important target crops for biotechnological innovation. Currently, there are more biotech traits available on the market in maize than in any other crop. The generation of transgenic plants is the crucial step in the development of new biotech trait products. For commercial applications, it is highly desirable to have a high throughput transformation system producing large number of high quality events in an elite genetic background. Transgenic maize production has made tremendous progress since the first successful report using the labor-intensive and time-consuming protoplast transformation method (Rhodes et al., 1988a). Development of microparticle bombardment transformation (Fromm et al., 1990; Gordon-Kamm et al., 1990) and *Agrobacterium*-mediated transformation (Ishida et al., 1996) technologies has made the generation of transgenic maize simpler and more reliable. Highly productive biolistic transformation systems were established in Hi-II with *BAR* as the selectable marker (Frame et al., 2000), and in the elite inbred line CG00526 with *PMI* as the selectable marker (Wright et al., 2001). Efficient *Agrobacterium*-mediated transformation systems were reported by using the inbred line A188 (Ishida et al., 1996; Negrotto et al., 2000), Hi-II (Zhao et al., 2001), and A188/Hi-II hybrids (Li et al., 2003). In the last few years, progress in genome engineering technologies has made it possible to make modifications and insert transgenes at specific chromosomal target sites in the maize genome (Shukla et al., 2009; Gao et al., 2010; Liang et al., 2014). Since assembly of TALEN and CRISPR-Cas9 nucleases can be carried out using standard molecular biology techniques, it is conceivable that their application in basic research for understanding the maize genome and in commercial trait development will progress rapidly in the coming years. This review will cover some of the aspects of maize transformation that are particularly important from a commercial trait development point of view. For sections covering transformation vector design, event quality and delivery of trait gene stacks, the discussions will be more general due to the limited number of maize-specific publications. However, the molecular approaches should be equally applicable to maize. The readers are also encouraged to read excellent review articles for more detailed information on general plant transformation systems (Hansen and Wright, 1999), mechanisms of *Agrobacterium*-mediated transformation (Gelvin, 2010), and maize tissue culture, regeneration and transformation (Jones, 2009; Wang et al., 2009).

## Development of maize transformation

The first method successfully used to transform maize cells employed the direct uptake of naked DNA into protoplasts, when stable transgene integration was demonstrated in calli derived from electroporated protoplasts of the Black Mexican Sweet (BMS) maize suspension cell line (Fromm et al., 1986). Two years passed before the first transgenic maize plant was reported (Rhodes et al., 1988a) because there was no efficient plant regeneration system. A simple and reliable regeneration system is a prerequisite for establishing an efficient transformation system. Out of the many studies in the early 1980s testing different starting materials including stem and immature embryos only a few actually reported success in regenerating maize plants from protoplasts (Rhodes et al., 1988b; Shillito et al., 1989). Immature embryos have proven to be an excellent source material to establish embryogenic callus and suspension cell cultures for preparing protoplasts for direct gene delivery (Rhodes et al., 1988b). Protoplasts were isolated from embryogenic cell suspension cultures derived from the A188 inbred line and an elite inbred line, and cultured on filter paper over a feed layer of BMS suspension cells to enable plant regeneration (Rhodes et al., 1988b). Protoplasts derived from A188 were successfully transformed by electroporation of a plasmid containing the *NPTII* (neomycin phosphotransferase) marker gene and resistant transformed calli were obtained on kanamycin selection media (Rhodes et al., 1988a). In the study, cell suspension cultures were initiated 18 months before transformation, as a result none of the regenerated plants produced viable seeds. Maize embryos may be induced to form one of the two major types of embryogenic calli: Type I calli with more compact structure having a group of embryos fused together or Type II calli with clusters of “friable” discrete single embryos (Armstrong et al., 1991; Hansen and Wright, 1999). To address the fertility issue of earlier studies Shillito et al. (1989) reported the use of Type II embryogenic calli derived from cultured immature embryos of an elite inbred line to generate embryogenic suspension cell cultures that can stay highly regenerable for several months. These cultures can then be harvested to isolate protoplasts for transformation. Omirulleh et al. (1993) reported the use of protoplasts from HE/89 cell suspension culture to generate transformants with the *NPTII* and *PAT* (phosphinothricin acetyltransferase) selectable markers. Wang et al. (2000) reported polyethylene glycol (PEG)-mediated transformation of protoplasts prepared from elite suspension cell lines using mannose selection, reaching transformation frequency of 0.06%.

Not long after the first reports of successful maize transformation using protoplasts were published, microparticle bombardment (also known as biolistic transformation) was successfully demonstrated to generate highly fertile maize transformants using embryogenic suspension cell cultures or calli as target tissue and the *BAR* (bialaphos resistance), *ALS* (acetolactate synthase), or *HPT* (hygromycin phosphotransferase) genes as selectable markers (Fromm et al., 1990; Gordon-Kamm et al., 1990; Walters et al., 1992; Vain et al., 1993). Compared with protoplast transformation events, biolistic transformation events obtained from embryogenic calli generally had much improved fertility. Later, Koziel et al. (1993) reported the use of immature embryos from an elite maize inbred as the target for biolistic transformation to introduce the *Bacillus thuringiensis* (Bt) *Cry1*Ab insecticidal gene with the *BAR* selectable marker. One of the described events (Bt176) was later launched as the first Bt maize product by Ciba-Geigy in 1996 (Table 1). Some other early commercial trait events that are still on the market were also generated using the biolistic transformation method (Table 1). Since the initial development there have been many improvements to the biolistic transformation technology. For example, Songstad et al. (1996) described an immature embryo biolistic transformation protocol using Hi-II germplasm and the *ALS* selectable maker. These authors also found that pre-culturing the immature embryos prior to bombardment greatly enhanced survival and transformation efficiency similar to results obtained with suspension cultures as targets (Vain et al., 1993). Brettschneider et al. (1997) and El-Itriby et al. (2003) also reported using conditioned immature embryos as targets of gene delivery, finding that transformation frequency was greatly increased when immature embryos were cultured before and after bombardment on medium with high osmolarity. In comparison with embryogenic callus cultures or suspension cells, the use of conditioned or cultured immature embryos as transformation targets greatly simplifies the target tissue preparation effort and at the same time shortens the transgenic plant production timeline. As a result, the transformation frequency and fertile transgenic plant production are both improved because the callus culture period is greatly shortened which lowers somaclonal variation. The drawback is that more greenhouse space is needed to grow stock plants as an immature embryo source. Also, immature embryo isolation itself is more time-consuming than callus subculture.

**Table 1:** **Transformation methods used to generate commercial maize transgenic events**.

**Event ID**	**Trait name**	**Trait(s)**	**Target variety**	**Gene delivery method**	**Selectable marker**	**Trait gene(s)**	**Source**
3272	Enogen™	Improve corn ethanol production by expedited starch liquefaction	NP2499 × NP2500	*Agrobacterium*	*PMI*	*Amylase*	a
5307	Agrisure® Duracade™	Coleopteran insect resistance	NP2222	*Agrobacterium*	*PMI*	*eCry3.1Ab*	b
98140	Optimum™ GAT™	Glyphosate tolerance	PHWVZ	*Agrobacterium*	*GAT*	*HRA (ALS), GAT*	c
Bt11	Agrisure™ CB/LL	Lepidopteran resistance; Glufosinate tolerance	BG	PEG-mediated protoplast transformation	*PAT*	*Cry1Ab, PAT*	d
Bt176	NaturGard KnockOut™, Maximizer™	Lepidopteran insect resistance	CG00526	Particle bombardment	*BAR*	*Cry1Ab, BAR*	e
DAS40278	Enlist™ Maize	AOPP herbicide tolerance	Hi-II	Silicon carbide whisker	*AAD-1*	*AAD-1*	f
DAS59122-7	Herculex™ RW	Coleopteran insect resistance; Glufosinate tolerance	Hi-II	*Agrobacterium*	*PAT*	*Cry34Ab1, Cry35Ab1, PAT*	g
DP-32138-1	32138 SPT maintainer	Fertility control for hybrid seed production	Unknown	*Agrobacterium*	*DsRed2*	*Ms45; alpha-amylase*	h
GA21	Roundup Ready™ Maize, Agrisure™ GT	Glyphosate tolerance	AT	Particle bombardment	*EPSPS*	Modified maize *EPSPS*	i
MIR162	Agrisure™ Viptera	Lepidopteran insect resistance	NP2500 × NP2499	*Agrobacterium*	*PMI*	*Vip3Aa*	j
MIR604	Agrisure™ RW	Coleopteran insect resistance	A188	*Agrobacterium*	*PMI*	*mCry3Aa*	k
MON810	YieldGard™	Lepidopteran insect resistance	Hi-II	Particle bombardment	*NPTII*, CP4 *EPSPS*	*Cry1Ab*	l
MON863	YieldGard™ Rootworm RW	Coleopteran insect resistance	A634	Particle bombardment	*NPTII*	*Cry3Bb*	m
MON87427	Roundup Ready™ Maize	Fertility control to eliminate the need for detasseling in hybrid seed production	LH198 × Hi-II	*Agrobacterium*	CP4 *EPSPS*	CP4 *EPSPS*	n
MON87460	Genuity® DroughtGard™	Drought stress tolerance	LH59	*Agrobacterium*	*NPTII*	*CspB*	o
MON88017	YieldGard™ VT™ Rootworm™ RR2	Glyphosate tolerance; Coleopteran insect control	LH198	*Agrobacterium*	CP4 *EPSPS*	CP4 *EPSPS, Cry3Bb1*	p
MON89034	YieldGard™ VT Pro™	Broad spectrum Lepidopteran resistance	LH172	*Agrobacterium*	*NPTII*	*Cry2Ab2, Cry1A.105*	q
MS3	InVigor™ Maize	Glufosinate tolerance, male sterility	H99	Electroporation	*BAR*	*BAR, Barnase*	r
MS6	InVigor™ Maize	Glufosinate tolerance, male sterility	H99	Particle bombardment	*BAR*	*BAR, Barnase*	s
NK603	Roundup Ready™ 2 Maize	Glyphosate tolerance	AW × CW	Particle bombardment	CP4 *EPSPS*	Double cassettes of CP4 *EPSPS*	t
T14	Liberty Link™ Maize	Glufosinate tolerance	He/89	PEG-mediated protoplast transformation	*PAT*	*PAT*	u
T25	Liberty Link™ Maize	Glufosinate tolerance	He/89	PEG-mediated protoplast transformation	*PAT*	*PAT*	u
TC1507	Herculex™ I, Herculex™ CB	Lepidopteran resistance; Glufosinate tolerance	Hi-II	Particle bombardment	*PAT*	*Cry1Fa, PAT*	v

Source:

a http://www.fda.gov/Food/FoodScienceResearch/Biotechnology/Submissions/ucm155608.htm

b http://www.aphis.usda.gov/brs/aphisdocs/10_33601p.pdf

c http://www.fda.gov/Food/FoodScienceResearch/Biotechnology/Submissions/ucm155603.htm

d http://www.fda.gov/Food/FoodScienceResearch/Biotechnology/Submissions/ucm161150.htm

e http://www.aphis.usda.gov/brs/aphisdocs2/94_31901p_com.pdf

f http://www.fda.gov/Food/FoodScienceResearch/Biotechnology/Submissions/ucm254647.htm

g http://www.fda.gov/Food/FoodScienceResearch/Biotechnology/Submissions/ucm155622.htm

h United States Patent Publication 8,257,930

i http://www.aphis.usda.gov/brs/aphisdocs2/97_09901p_com.pdf

j http://www.fda.gov/Food/FoodScienceResearch/Biotechnology/Submissions/ucm155598.htm

k United States Patent Publication 7,361,813

l http://www.fda.gov/Food/FoodScienceResearch/Biotechnology/Submissions/ucm161154.htm

m http://www.fda.gov/Food/FoodScienceResearch/Biotechnology/Submissions/ucm155785.htm

n http://www.fda.gov/Food/FoodScienceResearch/Biotechnology/Submissions/ucm304084.htm

o http://www.fda.gov/Food/FoodScienceResearch/Biotechnology/Submissions/ucm240080.htm

p http://www.fda.gov/Food/FoodScienceResearch/Biotechnology/Submissions/ucm155618.htm

q http://www.fda.gov/Food/FoodScienceResearch/Biotechnology/Submissions/ucm155607.htm

r http://www.aphis.usda.gov/brs/aphisdocs2/95_22801p_com.pdf

s http://www.fda.gov/Food/FoodScienceResearch/Biotechnology/Submissions/ucm155790.htm

t http://www.aphis.usda.gov/brs/aphisdocs2/000_01101p_com.pdf

u http://www.aphis.usda.gov/brs/aphisdocs2/94_35701p_com.pdf

v *http://www.aphis.usda.gov/brs/aphisdocs2/00_13601p_com.pdf*.

Other physical gene delivery methods have been developed for producing transgenic maize plants, including electroporation (D'Halluin et al., 1992), silicon carbide whisker-mediated transformation (Frame et al., 1994), and aerosol beam injector (Mets, 1993; Eby et al., 2004). To alleviate the requirement for Type II embryogenic calli or suspension cultures, D'Halluin et al. (1992) delivered DNA directly to immature embryos and Type I embryogenic calli through electroporation. Frame et al. (1994) described the development of the whisker-mediated maize transformation method which used embryogenic suspension cultures as target tissues and silicon carbide whiskers to deliver plasmid DNA. In this method, the mixture of suspension culture cells, DNA and whiskers is shaken vigorously, during which the whiskers presumably make tiny holes in the cells to allow DNA entry. Whisker-mediated transformation has successfully generated targeted gene insertion events at the *IPK1* locus mediated by zinc finger nucleases (ZFNs) using embryogenic cell cultures derived from Hi-II (Shukla et al., 2009). It was reported that aerosol beam injection of immature embryos from Stine elite inbred line 963 reached an average of around 3% transformation frequency which was better than the 1% frequency achieved via particle gun (Eby et al., 2004).

It was first demonstrated in the mid-1980s that *Agrobacterium* could mediate delivery of DNA into maize cells (Graves and Goldman, 1986; Grimsley et al., 1987). A few years later, Gould et al. (1991) reported successful transformation of maize using isolated shoot apices as target tissue. However, a routine method of *Agrobacterium*-mediated maize transformation was not developed until Ishida et al. (1996) described the use of immature embryos as the target tissue with an *Agrobacterium* strain containing a “super-binary” vector with *vir* genes from pTiBo542. Since then, the Ishida et al. (1996) method has been widely adopted by many labs around the world for transgenic maize production and it remains the method of choice for large scale event generation for trait research and commercial development. Many important factors affecting maize transformation, such as selection and regeneration conditions, already been established for biolistic transformation systems (Wright et al., 2001; Howe et al., 2002; El-Itriby et al., 2003), were readily optimized for *Agrobacterium*-mediated transformation. Several transgenic maize events produced by *Agrobacterium*-mediated transformation have been released to the market, some for more than 10 years already (Table 1).

High volume transgenic maize production via the standard *Agrobacterium* method requires a dedicated green house to supply a large number of zygotic embryos year-round. To overcome this greenhouse limitation, other maize transformation methods using alternative target tissues may be considered; however, the transformation efficiency is usually much lower. Biolistic transformation was attempted using apical meristems of immature embryos (Lowe et al., 1995) or shoot meristems directly derived from seedlings or multiple shoot cultures (Zhong et al., 1996). Transgenic plants able to transmit their traits through the germ-line could be recovered after multiplication of the bombarded shoot apical meristems using selection condition (Lowe et al., 1995). However, only chimeric plants with little or no transgenic germ-line cells were produced if the bombarded meristems did not go through further multiplication. After a biolistic transformation method with maize multiple shoot cultures was established (Zhong et al., 1996), the protocol was further demonstrated in and modified for more elite and hybrid lines from various genetic backgrounds. Li et al. (2002) also established an embryogenesis and multiple shoot clump system using shoot apical meristems of several elite maize inbred lines. They recovered *ALS* herbicide-resistant plants from microprojectile bombardment of multiple shoot clumps. O'Connor-Sánchez et al. (2002) developed an efficient biolistic transformation system for transgenic maize production with organogenic calli derived from shoot tips of 6 tropical and subtropical genotypes using phosphinothricin selection. Zhang et al. (2002) reported successful transformation of recalcitrant inbred lines B73 and PHTE4 by bombarding shoot meristematic cultures derived from germinated seedlings. Limited efforts were also reported for *Agrobacterium*-mediated transformation of shoot apical meristem cultures. Sairam et al. (2003) recovered transgenic maize plants through embryogenesis of meristem-derived calli after infection of shoot meristems with *Agrobacterium*. Kant et al. (2012) documented successful transformation of multiple shoot cultures of the CG62 inbred line with *Agrobacterium* strain EHA101. Despite these reports of successful shoot apex responses from various maize genotypes, transformation efficiency using either biolistic bombardment or *Agrobacterium* infection of shoot meristem cultures remains relatively low. Ahmadabadi et al. (2007) also developed a novel biolistic transformation method based on calli derived from immature maize leaf material from young seedlings. Al-Abed et al. (2006) reported the use of germinated split-seeds as explant for callus induction and regeneration in which the longitudinally split seed exposes 3 different tissues: the scutellum, the coleoptilar-ring and the shoot apical meristem. In this study, there was no molecular demonstration of successful production of transgenic plants using the split seed explants. However, Sidorov et al. (2006) reported successful *Agrobacterium*-mediated transformation of five different genotypes at frequency of 2 to 11% using callus tissues produced from longitudinally split nodal sections of 7 to 10-day old seedlings.

## Transgenic event selection

Effective selection is one of the most critical parts of an efficient transformation system. The presence of selection agent allows preferential proliferation of transformed cells and at the same time suppresses or kills untransformed cells. The ideal selection agent should not have a negative impact on subsequent regeneration, rooting and plant growth. Concurrent with the development of gene delivery methods, selectable marker optimization has been an integral part of developing efficient maize transformation systems. Both antibiotics and herbicides have been used widely as selection agents in maize transformation system development. Kanamycin (Fromm et al., 1986; Rhodes et al., 1988a; Lyznik et al., 1989) and hygromycin (Walters et al., 1992) are two of the earliest antibiotics used as selection agents in maize (Table 2). Table 2 lists commonly used selection systems to generate maize events.

**Table 2:** **Selection system for generating maize transgenic events**.

**Category**	**Selectable marker gene**	**Selection agent(s)**	**References**
Antibiotic resistance	*HPT*, hygromycin phosphotransferase	Hygromycin B	Walters et al., 1992
Antibiotic resistance	*NPTII*, neomycin phosphotransferase	Kanamycin, paromomycin, G418	Rhodes et al., 1988a; Xiayi et al., 1996; Zhang et al., 2003
Amino acid metabolism	*DAO1*, D-amino acid oxidase from *Rhodotorula gracilis*	D-alanine	Lai et al., 2007
Amino acid metabolism	*DSDA*, D-serine dehydratase (D-serine ammonia lyase) from *Escherichia coli*	D-serine	Lai et al., 2007; Lai et al., 2011
Herbicide resistance	*AAD-1*, aryloxyalkanoate dioxygenase from *Sphingobium herbicidivorans*	R-haloxyfop	Wright et al., 2010
Herbicide resistance	*ALS/AHAS*, maize acetolactate synthase/ acetohydroxy acid synthase	Chlorsulfuron, imazethapyr	Fromm et al., 1990; Lai et al., 2011
Herbicide resistance	*BAR*, phosphinothricin acetyltransferase from *Streptomyces hygroscopicus*; *PAT*, phosphinothricin acetyltransferase from *Streptomyces viridochromogenes*	Phosphinothricin, glufosinate, bialaphos	Fromm et al., 1990; Spencer et al., 1990; Omirulleh et al., 1993
Herbicide resistance	*EPSPS*, 5-enolpyruvoylshikimate- 3-phosphate synthase from *Agrobacterium* spp. CP4	Glyphosate	Heck et al., 2005
Herbicide resistance	*EPSPS*, 5-enolpyruvoylshikimate- 3-phosphate synthase from maize	Glyphosate	Howe et al., 2002
Herbicide resistance	*PPO*, protoporphyrinogen oxidase from *Arabidopsis*	Butafenacil	Li et al., 2003
Sugar metabolism	*PMI*, phosphomannose- isomerase (*man*A gene) from *E. coli*	Mannose	Negrotto et al., 2000; Wang et al., 2000; Wright et al., 2001

The herbicide selectable markers are often herbicide tolerance trait genes. Therefore, the use of an herbicide selectable marker is preferred in commercial transformation so the transgene can be easily monitored by herbicide application in subsequent introgressions. Several herbicide selection systems have been developed in maize including *BAR/PAT* (Fromm et al., 1990; Omirulleh et al., 1993), *ALS* (Fromm et al., 1990), *EPSPS* (Howe et al., 2002), *PPO* (Li et al., 2003), and *AAD-1* (Wright et al., 2010). Since the late-1990s several selectable marker systems have been developed to alleviate concern around the use of antibiotic or herbicide resistance genes, even though there is no evidence of horizontal transfer of these genes from transgenic plants to other sexually incompatible species. These new marker systems are based on sugar or amino acid metabolism (Table 2). Significant progress has been made toward development of so-called “positive” selectable marker systems for maize transformation (Sundar and Sakthivel, 2008). An excellent example of a positive selection marker is the phosphomannose isomerase (PMI) system. The application of these systems in maize is discussed in more detail below.

### *BAR* and *PAT* selectable marker

One of the first herbicide selectable marker genes used in maize transformation was *BAR* from *Streptomyces hygroscopicus* (Fromm et al., 1990; Gordon-Kamm et al., 1990; Spencer et al., 1990). Similar to *BAR*, the *PAT* gene from *Streptomyces viridochromogenes* also codes for phosphinothricin acetyltransferase activity (Omirulleh et al., 1993). Both *BAR* and *PAT* genes confer resistance to herbicide Liberty® which has glufosinate as its active ingredient. Koziel et al. (1993) described the generation of Event Bt176 expressing insecticidal protein *Cry1Ab* using the *BAR* selectable marker gene. Maize callus selection on bialaphos, conferred by either the *BAR* (bialaphos resistance) gene or the *PAT* (phosphinothricin acetyltransferase) gene, was found to be more efficient than selection on kanamycin (Register III et al., 1994). Subsequently, both *BAR* and *PAT* genes have been used in many studies (Ishida et al., 1996; Frame et al., 2002). Since it is relatively easy to achieve commercial level tolerance to herbicide Liberty® with *BAR or PAT* transgenic events, *BAR* and *PAT* have become the most commonly used herbicide resistance marker genes for commercial maize transformation.

### *EPSPS* and *GAT* selectable marker

Glyphosate resistance genes have been extensively explored as selectable markers since engineered glyphosate tolerance in crops is an important trait allowing control of a broad spectrum of weeds with the application of glyphosate herbicide. There are four major options to confer glyphosate tolerance: (1) the bacterial *aro*A genes encoding glyphosate-resistant 5-enolpyruvoylshikimate-3-phosphate synthase (EPSPS) such as from *Agrobacterium* spp. strain CP4, (2) the plant *EPSPS* genes encoding glyphosate-tolerant EPSPS mutants such as the maize EPSPS double mutant (T102I, P106S) present in event GA21 (Table 1), (3) the glyphosate oxidoreductase (*GOX*) gene, and (4) the glyphosate *N*-acetyltransferase (*GAT*) gene. Howe et al. (2002) described the use of glyphosate selection to select for maize transformants containing maize *EPSPS* mutant and *GOX* genes. Heck et al. (2005) described the production of commercial event NK603 (Table 1) by using CP4 *EPSPS* as a selectable marker and glyphosate as the selection agent. The GAT protein detoxifies glyphosate. Molecular evolution technology was used to improve *GAT* gene function and the resulting improved *GAT* variants enabled the regeneration of glyphosate tolerant transgenic maize plants (Castle et al., 2004). McCutchen et al. (2007) described the development of an improved *GAT* gene as a selection marker and showed that the *GAT* marker gave consistent performance with excellent transformation efficiency. In the side-by-side experiment to compare *GAT*, *BAR*, and *MO-PAT* (an optimized PAT gene with maize-preferred codons), *GAT* gave the best transformation frequency at about 64%, *BAR* at 34%, and *MO-PAT* at 30% (McCutchen et al., 2007).

### *ALS* or *AHAS* selectable marker

ALS (acetolactate synthase) is also known as AHAS (acetohydroxy acid synthase) and can be mutated at many different amino acid positions to confer various levels of tolerance to the numerous ALS inhibitor family of herbicides (Le et al., 2010). Several mutant *ALS* genes have been successfully employed as selectable markers in maize transformation. For example, the maize mutant *ALS* allele was used to generate transformants in one of the earliest reports of successful maize transformation with particle bombardment (Fromm et al., 1990). Howe et al. (2002) also described the use of *ALS* and chlorsulfuron to select for maize transformants. Lai et al. (2007) also described the use of an *AHAS* marker and compared the transformation efficiency of *AHAS* with *DSDA* and *DAO1* markers with three selection agents, Pursuit herbicide (active ingredient: imazethapyr), D-serine and D-alanine. They reported that average transformation frequencies of 31, 42, and 31% were obtained with Pursuit, D-serine and D-alanine, respectively.

### *PPO* selectable marker

A robust selectable marker system was developed based on the *Arabidopsis* protoporphyrinogen oxidase (PPO) gene coding for a double mutant (Y426M, S305L) resistant to the herbicidal compound butafenacil (Li et al., 2003). Maize transformants were produced using a flexible light regime to increase selection pressure. When the PPO enzyme activity is inhibited by butafenacil, accumulations of protoporphyrin IX causes light-dependent membrane damage (Lee et al., 1993). One advantage of *PPO* gene as a selectable marker is that it can generate a distinctive differentiating phenotype to identify putative transformants (Li et al., 2003). The selection system allows for efficient recovery of transformants using *Agrobacterium*-mediated transformation and multiple events tolerant to 3× effective field rate of butafenacil were obtained (Li et al., 2003). It should be noted that if other *PPO* mutants are to be used as selectable marker, the tolerance level of various mutants and the potency of different PPO enzyme inhibitors should be considered when designing experiments. Also, exposure of calli to lights should be limited during tissue transfer (Li and Nicholl, 2005).

### *AAD-1* selectable marker

Wright et al. (2010) isolated the *Rdp*A gene (also known as *AAD-1*) from *Sphingobium herbicidivorans* which encodes aryloxyalkanoate dioxygenase which cleaves the aryloxyphenoxypropionate (AOPP) herbicides including R-cyhalofop and R-quizalofop. These herbicides specifically inhibit the monomeric acetyl-CoA carboxylases from monocots. Wright et al. (2010) also described the use of the *AAD-1* gene as a selectable marker with R-haloxyfop as the selection agent for maize transformation. They produced *AAD-1* transgenic maize plants that were resistant to applications of the grass-active AOPP herbicides cyhalofop and quizalofop. Using *AAD-1*, the herbicide-tolerant maize event DAS40278 was developed into “Enlist™ Maize” (Table 1). It should be noted that *AAD-1* is homologous to the *tfd*A gene from soil bacterium *Ralstonia eutropha* and *tfdA* has been used to produce transgenic plants that are resistant to herbicide 2,4-dichlorophenoxyacetic acid or 2,4-D more than 20 years ago (Bayley et al., 1992).

### *PMI* selectable marker

Expression of *E. coli manA* gene encoding mannose-6-phosphate isomerase (PMI) enables efficient recovery of transgenic maize events on media containing mannose (Negrotto et al., 2000; Wang et al., 2000). A high throughput *Agrobacterium*-mediated transgenic maize event production system was established to meet the needs of trait research and development using the *PMI* selectable marker gene (Negrotto et al., 2000). Similarly, a highly efficient biolistic transformation system was developed in the elite variety CG00526 using embryogenic calli and mannose selection, which achieved an average transformation frequency of 45% (Wright et al., 2001). *PMI* enabled Syngenta to successfully develop several commercialized events including enhanced starch processing trait Enogen™ (Event 3272) and insect resistant trait events including Agrisure® RW (Event MIR604), Agrisure Viptera® (Event MIR162), and Agrisure Duracade™ (Event 5307) (Table 1). *PMI* has unique advantages for maize transformation including higher transformation frequency and powerful selection regimes. It takes less than a month to recover putative transformed callus which exhibits clear selection phenotypes. *PMI* is also a powerful tool to enable site-directed integration which depends on a reliable and efficient selectable marker (Que, 2006; Chen et al., 2014). Similar to *PMI*, another positive selection system based on the *xyl*A gene encoding xylose isomerase has been tested in maize, and xylose is used as the selection agent to identify transgenic events (Guo et al., 2007).

### *DAO1* and *DSD1* selectable marker

Two genes, *DAO1* and *DSDA*, encoding enzymes metabolizing D-amino acids have been studied as selectable markers. The *DAO1* gene from the yeast *Rhodotorula gracilis* encodes a D-amino acid oxidase (DAAO) which catalyzes the oxidative deamination of a range of D-amino acids (Erikson et al., 2004). Depending on the selection agent used, *DAO1* can be used either as a positive or negative selective marker (Erikson et al., 2004). For example, both D-alanine and D-serine are toxic to plants, but they are metabolized by DAAO into non-toxic products and are thus used as selection agents for transformation. The *E.coli DSDA* gene encodes a D-serine ammonia lyase which uses D-serine as the only substrate (Lai et al., 2011). Significant progress has been made in using the *DAO1* and *DSDA* selectable marker genes in maize transformation (Lai et al., 2007, 2011). Several vectors containing either the *DSDA* or the *DAO1* gene under the control of the maize ubiquitin promoter were tested for maize transformation frequency. Transformation frequencies of more than 20% were obtained using the *DSDA* marker with a concentration of D-serine between 5 to 15 mM (Lai et al., 2007). Similar high transformation frequency was obtained when *DAO1* gene was used as selection maker with D-serine or D-alanine as selection agent (Lai et al., 2007). These reports also compared the transformation efficiency of *DSDA* marker with another marker, the mutant *AHAS* gene and found that both markers produced similar maize transformation frequency (Lai et al., 2007, 2011).

### Optimization of selectable marker gene expression

Transformation frequency is affected significantly by the choice of marker gene expression cassette, including the use of expression components such as the promoter, Kozak sequence, 5'- and 3'- untranslated regions, the protein coding sequence and transit pepetides if the engineered protein must be delivered to the chloroplasts. Strong constitutive expression is usually desired to achieve high transformation efficiencies. However, the promoter effect can be dependent on the selectable marker gene. For example, Lai et al. (2007) observed no significant difference in transformation efficiency when *DAO1* gene was driven either by the sugarcane bacilliform virus (ScBV) or the maize ubiquitin promoter in both the hybrid and inbred lines. However, the *DSDA* selectable marker worked only under the control of the maize ubiquitin promoter, but not the ScBV or *AHAS* promoters. McCutchen et al. (2007) showed that the average transformation frequency using *GAT* driven by the maize ubiquitin promoter was higher when transcriptional enhancers were present. But use of different 35S enhancers, different enhancer combinations, and enhancer orientation had little effect on transformation frequency even though they increased GAT protein content and plant tolerance to glyphosate (McCutchen et al., 2007). However, selectable marker gene expression level may affect transgenic event quality. The percentage of single copy events produced using a construct without the 35S enhancer was only 38%, but it increased up to 88% when using constructs that contained the 35S enhancer (McCutchen et al., 2007). The effect of expression components other than the promoter is also important for achieving optimal transformation frequencies. For example, Howe et al. (2002) compared *EPSPS* expression cassettes with different introns in the 5'-UTR region and found that the first intron from the maize *HSP70* gene conferred higher transformation efficiency when compared to use of the first intron from the maize *ADH1* gene in the same position. However, Lai et al. (2007) did not observe a significant effect on maize transformation frequency when using the translation leader sequence PsFedi. This applied to both native and codon-optimized versions of the *DSDA* and *DAO1* marker genes. We also observed that addition of Kozak sequence to the codon-optimized *PMI* coding sequence did not further increase *PMI* transcript and protein levels (Que and Nicholl, 2012).

Since many of the selectable marker genes were originally isolated from bacteria, expression of these genes can be improved by optimizing their protein coding sequence to enhance transformation efficiency in plants. For example, Jayne et al. (2000) reported that a maize optimized *PAT* coding sequence resulted in higher transformation efficiency. Resistant tissues appeared earlier and grew faster. In the same report, a maize optimized *CAH* (cyanamide hydratase) coding sequence was also shown to enhance transformation efficiency. However, the effect of codon optimization on transformation frequency seems to be dependent on the transformation system. McCutchen et al. (2007) observed no difference in efficiency between *PAT* and *MO-PAT*. Also, the maize codon optimized *PMI* did not enhance transformation frequency even though the PMI protein level was increased more than 4-fold in transformed plants (Que and Nicholl, 2012). It is possible that the effect of *PMI* marker gene expression plateaus out once a sufficient level of PMI enzyme is reached to metabolize the toxic mannose present in the tissue. However, it is possible that the effect of gene expression on transformation frequency is highly dependent on crop and variety. We found that the same maize codon optimized *PMI* resulted in higher transformation efficiency in recalcitrant crops such as *indica* variety rice and sugarcane (Que and Nicholl, 2012).

## Tissue culture approaches to improve maize transformation

### Target variety and explant tissue

The ideal maize transformation line for trait research is an inbred line with excellent agronomic characteristics. The best tissue to start with should be easy to produce, inexpensive, available in large quantities year-round and highly transformable. The current transformation materials are far from ideal. Most labs use immature zygotic embryos or embryogenic calli derived from immature embryos for either biolistic or *Agrobacterium*- mediated transformation (Hansen and Wright, 1999; Jones, 2009). Immature embryos from several inbred genotypes have been successfully used to generate transgenic plants by *Agrobacterium*-mediated transformation methods (Gordon-Kamm et al., 2002; Huang et al., 2004; Huang and Wei, 2005; Frame et al., 2006; Hiei et al., 2006; Ishida et al., 2007; Valdez-Ortiz et al., 2007; Ombori et al., 2013). Some elite inbred lines have become the preferred genotypes for production of transgenic plants for trait development (Table 1). However, high efficiency *Agrobacterium*-mediated transformation is only achieved in a limited number of elite lines. Genotype dependency is a major constraint. Three key criteria contribute to this: competency of immature embryos to *Agrobacterium* infection, embryogenic callus formation and the ability to regenerate plants. The competency of immature embryos to *Agrobacterium* infection may be determined by cell defense response since two anti-apoptotic genes from baculovirus, *p35* and *iap*, had the ability to prevent the onset of apoptosis in response to *Agrobacterium* (Hansen, 2000). Many commonly used treatments to increase transformation efficiency such as heat shock, cold shock, antioxidants, and hypoxia may act through suppression of cellular response to *Agrobacterium* infection (Zhang et al., 2013).

Callus formation is another critical transformation factor. Typically, there are two types of embryogenic calli derived from maize immature embryos (Hansen and Wright, 1999; Jones, 2009; Wang et al., 2009). Immature embryos from many maize inbred genotypes produce Type I callus in response to induction and are more difficult to transform. Through media component optimization *Agrobacterium*-mediated transformation of inbred genotypes that produces Type I callus has been substantially improved (Gordon-Kamm et al., 2002; Huang and Wei, 2005; Ishida et al., 2007; Valdez-Ortiz et al., 2007). Some transformation studies use Type I callus (Wan et al., 1995; Wang et al., 2009), but most studies use Type II callus. Type II callus is derived from the immature embryo scutellum. It is friable, fast growing and highly regenerable (Armstrong et al., 1991). These characteristics favor transgenic plant selection and regeneration, thus the friable Type II callus and its liquid suspension were early maize transformation target tissues (Fromm et al., 1990; Gordon-Kamm et al., 1990; Walters et al., 1992; Frame et al., 1994). However, the formation of Type II callus is highly genotype-dependent and is limited to a few genotypes such as A188, Hi-II, A188 × Hi-II, B73, and Cz643 (Green and Philips, 1975; Armstrong and Green, 1985; Armstrong et al., 1991; Frame et al., 2002; El-Itriby et al., 2003; Ombori et al., 2008). Among the genotypes producing Type II callus, Hi-II, a hybrid line, is the most commonly used because of its reliable and high transformation efficiency in laboratories. There is considerable interest and effort to identify the genetic loci involved in the embryogenic callus formation. The idea is to use these loci to make non-responsive inbred lines transformable (Jones, 2009). Hi-II is a poor line to evaluate agronomic traits such as yield improvement and abiotic stress tolerance because it has low agronomic performance and a non-uniform genetic background. Also, a consistent, differential performance between two distinct sources of Hi-II germplasm (PTFHT and WHT) in biolistic-mediated transformation has been reported (Wang et al., 2009). The transformation frequency was four times higher in WHT compared to PTFHT when used in biolistic transformation, but was not significantly different when used in *Agrobacterium*-mediated transformation (Wang et al., 2009).

### Optimization of media components

Optimized culture media is indispensable for establishment of an efficient transformation system. For most maize genotypes, N6, MS, or LS salt-based media are used at various stages of the transformation process. In order to achieve optimal transformation of a specific genotype using a particular selectable marker system, it is necessary to optimize media components such as carbon source, vitamins, amino acids, and plant growth regulators. For example, when using MS and N6 salts lower nitrate and high ammonium levels are known to promote induction of compact Type I callus, whereas higher nitrate levels produce Type II callus (Elkonin and Pakhomova, 2000). Frame et al. (2006) described the advantages of using MS and N6 salts to improve transformation efficiency in three maize inbred lines. Studies have shown that addition of antioxidants such as dithiothreitol (DTT), L-cysteine or low salts either alone, or combined, in the co-cultivation medium was shown to enhance T-DNA transfer and improve transformation efficiency (Frame et al., 2006; Vega et al., 2008; Yu et al., 2013).

Plant growth regulators play a critical role in different phases of maize transformation. 2,4-D and dicamba are often used to induce formation of regenerable callus. Dicamba is considered the most effective across many varieties (Duncan et al., 1985). Addition of the ethylene biosynthesis inhibitor silver nitrate to co-cultivation and callus induction media containing 2,4-D, casamino acids and proline increased the production of Type II callus from immature embryo cultures derived from genotypes such as Hi-II (Armstrong et al., 1991; Songstad et al., 1991; El-Itriby et al., 2003). Efficient shoot regeneration is achieved when regeneration medium is supplemented with individual or combinations of several cytokinins such as 6-benzylaminopurine (BA), kinetin and thidiazuron (TDZ). Zhao et al. (2008) report that 2.22 μM BA in combination with 4.64 μM kinetin was optimal for efficient regeneration from mature embryo cultures from four inbred lines. In addition, the antibiotics used in *Agrobacterium*-mediated transformation experiments can greatly affect callus growth. For example, Cheng et al. (2004) reported a 3-fold reduction in Hi-II callus induction when 50 or 250 mg/L cefotaxime was compared to the addition of 100 mg/L carbenicillin.

### Application of automation to increase process efficiency

Current transformation technology involves time-consuming and laborious tissue culture processes. Because labor is the largest cost associated with transgenic event generation, there has been a concerted effort to reduce the time and labor required for each step. In the last 30 years, automation has been explored to reduce labor, physical injury to explants during transfer and physiological stress during culture. As early as in 1985, Tisserat and Vandercook (1985) developed an automated plant culture system prototype which used computer control to introduce or replenish plant tissue culture media in a sterile environment. Rout et al. (2013) described a simple and rapid protocol that combined liquid media, efficient plant-handling procedures with simplified media and culture steps. They transformed and regenerated monocots in as little as 4–8 weeks. The advantages of this method are shorter production time, higher throughput, lower material and labor costs, and ergonomic safety benefits. In addition, transformation frequency was maintained or even increased (Rout et al., 2013).

The cost and labor associated with immature embryo isolation is significant especially when large numbers are required to generate hundreds and occasionally thousands of transgenic events. Here embryo supply and excision are bottlenecks. Manual embryo excision also poses ergonomic injury risks when large numbers are required. A simple, robust, consistent and high throughput method to isolate immature maize embryos is needed. Several publications describe methods and devices to rapidly isolate and transfer maize embryos. Adams et al. (2005) described an apparatus to isolate maize embryos by washing a mixture of embryos and endosperms with a liquid jet stream and then passing the mixture through a sterile mesh. The small embryos and some endosperm debris were collected and further processed to remove debris. Embryos isolated by this method were still transformable, albeit with lower transformation frequency in the example provided. Davis and Mann (2006) developed a simple method to isolate immature maize embryos using a vacuum device. The partial or intact immature embryos were useful for callus production and fertile plant regeneration. Bullock (2011) described an apparatus to isolate immature maize embryos in bulk using pressure liquid stream. Barreiro et al. (2011) described an apparatus to isolate immature maize embryos by centrifugation. A challenge with mechanical isolation is cross-contamination. Microbial contamination from even one ear can ruin an entire batch of embryos. Timmis et al. (2004) described a complex, automated, system that included robots to isolate individual plant embryos. The modules in this system include a separation module to separate plant embryos from the embryogenic mass and sort them by size. Swanda et al. (2013) described the further development of this system by including a drying module and using robotic arm to transport the plant embryos between modules in a predetermined sequence. Adoption of a similar system should be very beneficial for increasing productivity in large scale maize transformation facilities.

## Transformation vector design and transgene expression

For commercial transformation detailed vector design is required to ensure efficient transgenic event production, desirable trait efficacy and timely product regulatory approval and registration. Transgene expression level *in planta* is critical to ensure trait efficacy. Too little expression of intended proteins can affect trait performance by not conferring the desired trait efficacy. Low trait protein expression may reduce trait performance, and excessive expression may reduce plant performance. Trait gene and T-DNA design should meet several criteria. All unwanted or putative open reading frames should be minimized by inserting a translation stop codon or altering a translation start codon. Protein sequence identical to or with significant homology to toxins and allergens must be eliminated (Ladics et al., 2007; Silvanovich et al., 2009; Harper et al., 2012). The trait gene should have the optimal promoter and Kozak sequence (Luerhsen and Walbot, 1994; Joshi et al., 1997). Codon optimization may be required to produce the trait protein. The position and location of trait genes within the T-DNA can influence trait gene performance. The plant selectable marker should be adjacent to the T-DNA Left Border (LB) to increase recovery of events with full-length inserts. T-DNA insertion position within the plant genome can also affect transgene expression since regulatory elements adjacent to the T-DNA insert may either activate or repress transgene expression (De Buck et al., 2013). If possible, insulators or enhancer blocking sequences can be included to minimize interference by nearby endogenous enhancers and chromatin-mediated silencing (Hily et al., 2009; Singer et al., 2011).

Molecular stack vectors, which contain several trait genes, require tactics to mitigate interference between the individual genes (Que et al., 2010). In addition to the product safety and event quality requirements, several steps may improve the production of high quality molecular stack events. (1) Validate the function of each trait gene by itself before stacking with other genes; (2) Minimize the use of promoters with viral enhancers; (3) Remove structures such as direct or inverted repeats that can potentially affect construct stability or result in the generation of unpredicted transcripts such as small RNAs that can affect either transgene or endogenous gene expression (Goodarzi et al., 2012); (4) Consider producing several variants of the molecular stack to explore the influence of trait gene cassette position and orientation on trait gene efficacy; and (5) Include insulator or enhancer blocking sequence to minimize interference by the adjacent cassettes (Gudynaite-Savitch et al., 2009; She et al., 2010; Singer et al., 2011).

## Improvement in transgenic event quality

### Backbone reduction

*Agrobacterium* can efficiently transfer a small number of copies of relatively large segments of T-DNA to plant cells with minimal rearrangement in a well-documented process (Sheng and Citovsky, 1996). However, non-T-DNA vector backbone sequence from the transformation plasmid is present in transgenic events (Martineau et al., 1994), up to 75% in one study involving tobacco (Kononov et al., 1997). In many cases, the backbone sequence in transformed events is linked to the T-DNA across one or both the Left Border (LB) and Right Border (RB). In extreme cases, the entire vector is integrated into the transformed plant genome (Kononov et al., 1997). Vector backbone sequence might negatively impact transgene expression and stability. Also, the presence of backbone sequence complicates transgenic trait product development because additional studies will be required to satisfy product safety standards. This takes time and increases costs. High quality transgenic events contain a single-copy of the complete, intact T-DNA and have no vector backbone. Various backbone reduction approaches have been explored to accomplish this goal.

#### Incorporation of negative selection gene cassette in backbone

To reduce the number of backbone integration events, Hanson et al. (1999) placed a barnase gene expression cassette in the non-T-DNA portion of a binary vector. The barnase coding sequence contained an artificial intron and is lethal to plant cells that acquired the vector backbone. This reduced backbone presence by more than 30% in several plants including tobacco, tomato, and grape. Alternatively, Stuiver et al. (2000) used a gene silencing cassette targeting plant housekeeping genes. Ye et al. (2008) used expression cassettes encoding genes that inhibit plant development, including bacterial levansucrase (*sac*B), maize cytokinin oxidase (*CKX*), *Phaseolus* GA 2-oxidase (*GA2-ox*), and bacterial phytoene synthase (*crt*B). The lowest frequency of backbone presence in soybean was observed with a constitutively expressed *CKX* gene, followed by *crt*B (Ye et al., 2008). Short backbone fragments still present in some events may be caused by LB read through and termination within the backbone reduction gene. Overall the frequency of transgenic soybean plants containing one or two T-DNA copies and no vector backbone significantly increased when the *CKX* or *crt*B backbone reduction vectors were used.

#### Use of multiple left borders in T-DNA

Kuraya et al. (2004) inserted two to three additional T-DNA LB copies near the original T-DNA LB. They hypothesized that inefficient termination of T-DNA intermediate was responsible for backbone insertion, and that the additional LB sequence would suppress this. They claim their approach is easier than placing a “lethal gene” outside the T-DNA and that it will efficiently produce backbone-free events.

#### Launching T-DNA from agrobacterium chromosome

Oltmanns et al. (2010) reported that launching the T-DNA from the *Agrobacterium pic*A chromosome locus increased the frequency of single transgene integration events and almost eliminated vector backbone presence in both *Arabidopsis* and maize. The drawback is that construction of vectors that insert into the *Agrobacterium* chromosome is more complicated than normal binary vectors. Also, the overall transformation efficiency is much lower. Both factors may limit the practical application of this approach.

#### Use of binary vector with replication origin from pRi

Most labs use binary vectors based on a broad host range origins of replication RK2 oriV (IncPa) and pVS1 ori for *Agrobacterium*-mediated crop transformation (Hellens et al., 2000; Lee and Gelvin, 2008). Vector systems based on pSa (IncW) and pRi origins of replication that function in *Agrobacterium tumefaciens* were also used in transformation (Lee and Gelvin, 2008). For example, a binary vector containing the pRi ori from *Agrobacterium rhizogenes* Ri plasmid was used to make a plant genomic DNA library with a 23.6 Kb mean insert size (Simoens et al., 1986). However, pRi ori-based binary vectors are not routinely used due to its low copy numbers and thus require more effort for molecular manipulation. Ye et al. (2011) reported that pRi ori binary vectors significantly increased the frequency of single-copy, backbone-free transgenic events relative to RK2 oriV-based binary vectors in multiple crops, including maize.

### Transgene copy number and rearrangements

Transgenic plants that contain a single copy transgene are often preferable to those with multiple copies. Transgenes present in high copy numbers have been associated with functional and structural instability (Meyer and Saedler, 1996). A complex integration pattern also makes structural characterization more difficult. In biolistic transformation, use of a DNA fragment containing only the expression cassettes instead of whole plasmids resulted in simple transgene inserts (Fu et al., 2000). Large scale studies verified that low copy events occurred more frequently when low DNA doses were bombarded even though transformation efficiency was generally lower (Lowe et al., 2009). Srivastava and Ow (2001) reported that the Cre-*lox* recombination system generated single-copy transgenic plants by Cre-mediated resolution of multi-insert events. Here, biolistic transformation was used to co-deliver a Cre-expressing vector and a transgene construct flanked by inverted *lox* sites to maize cells. A high percentage of the primary transformants had a single copy transgene. In addition, 60% of the single copy plants lacked the recombinase gene, resulting in an overall low copy transformation efficiency of 23%. It is likely that transiently expressed Cre recombinase removed the extra transgene copies. In place of co-transformation, the recombinase gene can also be crossed into and out of transgenic events. Alternatively, the recombinase can be expressed using an inducible promoter so that it deletes itself from the genome. This was demonstrated using the R/*RS* system and the Cre-*lox* system (Sugita et al., 2000; Zuo et al., 2001). Sugita et al. (2000) used the GST-MAT vector to generate high frequency, marker-free transgenic plants containing a single transgene. They fused the *Zygosaccharomyces rouxii* R recombinase gene to the safener inducible maize glutathione-S-transferase (GST-II-27) promoter. Safener induction removed the isopentenyltransferase (*ipt*) selectable marker gene. Kondrak et al. (2006) assessed use of the R/*RS* recombination system for generating marker- and backbone-free transgenic plants. Their vector constitutively expressed a plant-adapted R recombinase and a *codA*-*NPTII* bi-functional, positive/negative selectable marker gene. It only has the T-DNA right border so the whole plasmid is inserted into the plant genome. The R recombinase recognition sites (*RS*) are positioned to recombine and delete both the bi-functional marker genes and the binary vector backbone, leaving only the trait gene flanked by the *RS* site and RB sites. For the *Agrobacterium*-mediated transformation system there is still no simple and effective molecular approach to control transgene copy number other than that described above in using site-specific recombination.

### Intactness of transgene insert

Another factor affecting transgenic event quality is the transgene intactness. Direct physical DNA delivery methods such as particle bombardment do not have a known size limit. BAC vectors comprising minichromosomes over 100 Kb in length have been delivered by particle bombardment (Carlson et al., 2007; Ananiev et al., 2009). However, large DNA molecules are more difficult to manipulate and they are subject to shearing during isolation, purification and processing for transformation. Foreign DNAs may also be more susceptible to nucleases since they are not protected in nucleosomes or chromatin. Protecting foreign DNA during delivery may enhance transformation frequency and improve transgene intactness. Sivamani et al. (2009) reported a 3.3-fold increase in stable transformation efficiency by coating the DNA with protamine. Co-precipitating plasmid DNA with protamine presumably forms stable nano-complexes. *In vitro* assays showed this new coating method protected plasmid DNA from DNase degradation and kept plasmid DNA intact much longer than spermidine-plasmid DNA complexes when exposed to rice cell extract. The Hansen and Chilton (1996) “Agrolistic” method used transiently expressed *Agrobacterium virD* to simplify the transgene integration pattern and reduce transgene copy number in biolistic transformation events. Recently, Ziemienowicz et al. (2012) obtained a high percentage of intact transgene insertions in transformed *Triticale* microspores using an *in vitro* assembled nano-complex of T-DNA, VirD2, RecA, and Tat2 cell-penetrating peptide. Tat2-mediated transformation also produced more low copy events that gave improved transgene expression. These new methods may increase transgene intactness in maize biolistic transformation events.

Sequence analysis of T-DNA insert junctions in *Agrobacterium*-mediated transformation events suggests that T-DNA integration may be guided by microhomology between the T-DNA strand ends and the chromosomal breaks via strand invasion or by the micro-homology between the ends of the replicated T-DNA strands and the chromosomal breaks via non-homologous end joining (see review by Gelvin, 2010). These studies also indicate that internal T-DNA sequence was mostly intact, and the T-DNA ends were resected to various degrees. The T-DNA RB is protected by covalently-bound VirD2 during transfer into the nucleus which may remain there until integration and deletions in the RB-proximal region are usually much smaller than those at the LB-proximal region (Tinland et al., 1995). Therefore, the selectable marker gene cassette is usually placed next to the LB to increase the likelihood that events containing all the T-DNA gene cassettes are recovered. However, this design merely enriches for events with an intact selectable marker gene cassette and does not increase the overall number of transgenic events with intact inserts. Use of supervirulent *Agrobacterium* strains containing helper plasmids with additional copies of virulence genes such as *virG* and *virE* may enhance transgene delivery and increase both the number and percent of events with intact T-DNA inserts (Frary and Hamilton, 2001). Also, even though the use of *recA+ Agrobacterium* strains may give significantly higher transformation efficiencies for smaller size T-DNA vectors (Frary and Hamilton, 2001), the use of *recA*-deficient strain resulted in higher transformation efficiency for a 150 Kb T-DNA vector (Hamilton et al., 1996). In fact, *recA*-deficient *Agrobacterium* strain is necessary to maintain structural stability by reducing intramolecular rearrangement if repetitive sequences are present within the T-DNA.

## Conventional approaches to deliver trait gene stacks

Trait gene stacking promises to improve pest management, disease control and abiotic stress resistance. In maize, transgenic traits for insect control and weed management typically incorporate two or more modes of action (Que et al., 2010). Most transgenic maize products on the market contain a breeding stack of existing events containing 1–2 trait genes each. Breeding will continue to be an indispensable method to introgress transgenic traits into commercial germplasm, but stacking traits this way is time-consuming and expensive. Therefore, it is preferable to limit the number of transgenic loci to no more than 3–4 and to include multiple trait genes in each DNA construct for transformation. A molecular stack is a single transformation construct containing several trait genes. As the trait gene number increases, new tools will be needed to efficiently transform molecular stacks.

### Large DNA molecule delivery

The basic strategy is to use DNA fragments or binary vectors with several linked trait genes in biolistic- and *Agrobacterium*- mediated transformation. Because transgenes tend to insert at a single locus, there is a high probability that intact, single-copy events will be generated. But DNA size presents challenges. When introduced using biolistics, large DNA fragments may be sheared during preparation, delivery or integration into the plant genome. Large T-DNAs reduce the transformation frequency of *Agrobacterium*-mediated methods. Generally, transformation frequency decreases as the T-DNA size increases (Park et al., 2000). Reports of efficient transformation using binary vectors with T-DNAs larger than 50 Kb are not common in monocot crop plants, especially maize. Hamilton et al. (1996) developed the BIBAC vector, a low copy plasmid in both *E. coli* and *Agrobacterium* to transform large T-DNAs. They also used a supervirulent *Agrobacterium* strain containing a helper plasmid with additional copies of *virG* and *virE* to enhance transgene delivery. Frary and Hamilton (2001) systematically compared the transformation efficiency of three BIBAC vectors with 7 Kb, 30 Kb and 150 Kb T-DNAs and different *Agrobacterium* strains in a moderately transformable tomato line. They found that the helper plasmid with extra copies of *virG* was absolutely required to successfully introduce the 150 Kb T-DNA. Also, additional *virE* copies in the helper plasmid increased transformation frequency. Finally, a 150 Kb T-DNA vector produced much lower number of transgenic tomato plants with intact T-DNA copies compared to a 30 Kb T-DNA vector (Frary and Hamilton, 2001). Liu et al. (1999) showed that the transformation efficiency of a transformation-competent artificial chromosome (TAC) vector with 80 Kb T-DNA insert was about 30% lower on average compared to a vector with 4.4 Kb insert in an easily transformable model plant *Arabidopsis*. We recently found that a 120 Kb T-DNA insert vector produced a 10-fold decrease in maize transformation efficiency of events with intact T-DNA inserts compared to a 15 Kb T-DNA vector (Defontes et al., unpublished results). The cause for the low efficiency was not analyzed systematically. It is possible that multiple factors contribute to this. It could be the T-DNA processing, transfer or integration step. It is generally accepted that *Agrobacterium* delivers VirE2 protein separately from the T-DNA using the VirB/D4 Type IV secretion system. Then VirE2 associates with the T-DNA in the plant cell cytoplasm to form the mature T-complex (Gelvin, 2010). Perhaps large T-DNA transfer prematurely terminates or limited VirE2 renders the large T-DNA more susceptible to degradation by plant cell nucleases.

### Co-integration

An alternative to trait gene stacking is simultaneously introducing multiple vectors containing one or more transgenes. This is co-transformation, in which unlinked transgenes from different vectors are randomly inserted in either linked or unlinked loci (Spencer et al., 1990). It is simple and efficient to co-integrate multiple DNA fragments or plasmids at a single locus using biolistic transformation. In maize the transformed locus is stably inherited (Register III et al., 1994; Zhong et al., 1996). Co-integration of T-DNAs using *Agrobacterium*-mediated transformation has also been reported. The approaches include co-infection with *Agrobacterium* strains harboring different binary vectors (Depicker et al., 1985; McKnight et al., 1987), infection with *Agrobacterium* containing two distinct binary vectors (De Framond et al., 1986) and infection with *Agrobacterium* containing a single binary vector with two separate T-DNAs (Komari et al., 1996; Miller et al., 2002; Huang et al., 2004).

## Site-directed genome modification and targeted transgene insertion

### Targeted genome mutagenesis

A simple, fast and reliable system to generate mutations in target sequences at the genome level is necessary to study gene function. As in other plants, targeted mutagenesis is very low and impractical in maize without artificial induction of double-stranded breaks at the target region and thus is not suitable for generating targeted mutations. Other methods such as transposon-mediated mutagenesis with the endogenous *Mutator* (*Mu*) and *Activator* (*Ac*) elements, and TILLING (Targeting Induced Local Lesions IN Genomes) have been used to generate mutations in maize genes for many years (May et al., 2003). These methods generate random mutations that typically require a dedicated facility to support large population screens (Till et al., 2004). When successful these methods produce a limited number of alleles at the target gene. Ideally it should be possible to precisely manipulate target genes in the genome. This requires tools that can recognize a specific DNA sequence and induce the intended change. In maize this was first achieved through the use of RNA/DNA chimeric oligonucleotides (Zhu et al., 1999). Here, two independent target sequences within the endogenous *AHAS* gene were modified, and the mutants were identified because they confer resistance to either imidazolinone or sulfonylurea herbicides. In a subsequent report, Zhu et al. (2000) showed the imidazolinone-resistant maize plants stably inherited and expressed mutant *AHAS* allele. However, the gene conversion efficiency was about 10^−4^, not suitable for routine applications in genes for which mutations are not efficiently identified with a selection agent.

The last few years produced several technologies to design and construct site-directed nucleases that can specificity recognize a long DNA sequence such as ZFNs, engineered meganucleases, TAL effector nucleases and CRISPR-Cas9 nucleases (Voytas, 2013; Puchta and Fauser, 2014). The CRISPR-Cas9-based system is particularly attractive for mutagenesis because the mutagenesis frequency is usually quite high; Also, the target specificity is programmed by the 20-bp guide RNA molecule, and multiple sequences can be targeted for mutagenesis at the same time (Belhaj et al., 2013). Shukla et al. (2009) reported the successful use of 4 pairs of engineered zinc-finger nuclease cleaving the maize *IPK1* gene. Gao et al. (2010) achieved high frequency mutagenesis using a redesigned homing endonuclease I-*Cre*I to target an engineered meganuclease to sequence adjacent to the *LIGULELESS1* (*LG1*) gene promoter. They used *Agrobacterium*-mediated transformation of immature embryos, and produced monoallic, biallelic, and chimeric mutations at the *LG1* locus in ~3% of the T0 transgenic events. Djukanovic et al. (2013) reported the mutagenesis of the *MS26* fertility gene. They evaluated three different engineered meganucleases, and found the mutagenesis frequency in stably transformed plants correlated very well with transient assay data in maize BMS cells and in human HEK 293 cells with transgenic target sequences. Ems26++, the most active variant produced a 5.8% mutation frequency at the *MS26* site in the T0 transgenic plants. One of the 21 transgenic lines contained a biallelic mutation and had the expected male sterile phenotype. Liang et al. (2014) reported targeted mutagenesis in maize using TALENs and the CRISPR/Cas9 system. Five TALENs were used to target *ZmPDS, ZmIPK1A, ZmIPK, and ZmMRP4* gene sequences. In transient protoplast assays, TALENs for the *ZmIPK* and *ZmMRP4* sequences cleaved their targets in 9.1% and 23.1% of the cells, respectively. Cleavage efficiencies were much lower for the three TALENs targeting *ZmPDS* and *ZmIPK1A*. About 39.1% of the stable T0 transformants made with *ZmIPK* TALENs using *Agrobacterium*-mediated transformation contained somatic mutations in the target gene. They did not report how many transformants contain heritable germline mutations. They also used the CRISPR/Cas9 system to induce mutations in a protoplast transient assay, and showed that co-expressing the Cas9 protein plus two guide RNAs induced targeted mutations at the *ZmIPK* target sequence. Targeting the same gene, the CRISPR/Cas9 system produced estimated 13.1% mutation efficiency and the TALENs 9.1% efficiency (Liang et al., 2014). The above reports indicated that the available site-directed nucleases can be designed to cleave specific sites in the maize genome. These new tools enable functional dissection of maize genes at the nucleotide level, without changing their genomic context. Each engineered nuclease has its pros and cons. Their commercial trait development potential will be influenced by the way regulatory agencies define the products made with these new site-directed nucleases. Some consider this technology to be GM in nature, while others consider it to be non-GM.

### Targeted integration mediated by site-directed nucleases

Another approach to create multi-gene stacks is targeted integration of new transgenes to predetermined genomic locus. One method relies on targeted integration mediated by homologous recombination between the donor DNA molecule and the chromosomal locus. However, targeted transgene insertion by homologous recombination occurs at low frequency in higher plants. The introduction of meganuclease I-*Sce*I (Puchta et al., 1996) which produces chromosomal breaks made this method more effective. This was demonstrated by restoring a promoterless *BAR* gene in maize suspension cells using a codon-optimized I-*Sce*I gene (D'Halluin et al., 2008). The target lines were created using the highly transformable HE/89 genotype, which facilitated the evaluation of different conditions such as the gene delivery method and target site effect. The study revealed significant variation in targeted integration frequency among different target lines. Also, donor molecule delivery by particle bombardment produced a higher targeting frequency compared to *Agrobacterium*-mediated delivery using the same target line (D'Halluin et al., 2008).

Our targeted insertion approach in maize was similar, except that meganuclease I-*Ceu*I was used to produce targeted transgene insertion in rice and maize loci (landing pads). The selection strategy restored function of a truncated *PMI* marker gene by homologous recombination (Que, 2006). *Agrobacterium*-mediated transformation delivered the I-*Ceu*I expression vector and targeting donor vectors into target lines harboring truncated selectable marker *PMI* gene. Targeted insertion events were recovered at low efficiency because A188 × Hi-II progeny were being retransformed and were likely segregating for transformation competence (Que, 2006). A highly transformable inbred line would likely increase recovery of targeted insertion events. We recently reported TALEN-mediated transgene insertion at target loci by reconstituting a truncated *PMI* selectable marker gene using efficient *Agrobacterium*-mediated transformation of an elite maize inbred (Chen et al., 2014).

Ayer et al. (2013) addressed the low maize transformation efficiency issue by using somatic ectopic recombination between the donor and transgenic target loci. Here, the target locus had an I-*Sce*I cleavage site and the donor locus had the complementing *NPTII* gene flanked by homologous regions and two I-*Sce*I sites. The donor locus also had a dexamethasone-induced expression cassette for an I-*Sce*I protein fused to the rat glucocorticoid receptor (GR) domain (I-*Sce*I::GR). Donor excision occurred in F1 plants that contain both the transgenic target and donor loci. These plants were selfed, but no germline kanamycin-resistant recombinants were recovered in in the F2 progeny despite the presence of numerous somatic recombination sectors in F2 plants. They used F2 immature embryos to produce callus on kanamycin media, and several plants that contained targeted insertion were regenerated. However, the targeted insertion efficiency was less than 1%. This may be due to a relatively low rate of target locus cleavage caused by limited dexamethasone absorption or low expression I-*Sce*I::GR fusion protein in maize (Ayer et al., 2013).

In a related approach, Ainley et al. (2013) used engineered zinc-finger nucleases (ZFNs) to mediate targeted trait gene insertion at the existing transgene loci that contained cleavage sites for ZFNs. Transgenic lines with an artificial transgene landing pad consisting of ZFN recognition sites and fixed flanking sequence were created (Ainley et al., 2013). They used biolistic transformation to deliver the ZFN expression and target vectors to immature embryos hemizygous for the transgenic landing pad locus. Successful integration of the *AAD-1* herbicide tolerance gene cassette at the landing pad locus containing a *PAT* transgene was achieved at about 3% efficiency (Ainley et al., 2013). Targeted integration efficiency differed significantly between the 4 different ZFNs they tested. It also significantly differed between the two landing pad lines, suggesting targeted integration may be influenced by genome location as observed by D'Halluin et al. (2008).

Breakthroughs with engineered nucleases made it practical to design nucleases that mediate targeted insertion into specific plant genome locations. Shukla et al. (2009) successfully inserted a *PAT* transgene into the maize *IPK1*gene by homologous recombination using four pairs of engineered ZFNs. The ZFN and *PAT* vectors were co-delivered by whisker-mediated transformation of cultured maize cells. They show that 815 base pair (bp) of homologous flanking sequence is sufficient to guide the homologous recombination. Southern blot analyses indicated the targeted insertion events had no random insertions of either the ZFN or *PAT* vectors, whereas non-targeted events contained many random insertions. They found little to no insertion activity at the *IPK2* gene which is highly homologous to *IPK1*, suggesting the approach is highly specific (Shukla et al., 2009).

Other engineered nucleases have been successfully used for targeted mutagenesis in maize (Gao et al., 2010; Djukanovic et al., 2013; Liang et al., 2014), and may be good candidates to mediate targeted transgene insertion at the desirable chromosomal loci. D'Halluin et al. (2013) used an engineered meganuclease to mediate targeted insertion at a site adjacent to a transgene locus in cotton. We have also recently demonstrated targeted transgene insertion to a native chromosomal locus in an elite maize inbred with three different site-directed nuclease technologies, TALEN, engineered meganuclease and CRISPR-Cas9, using either biolistic or *Agrobacterium*-mediated delivery (Chen et al., unpublished results).

Each of the available engineered nuclease technologies has its strengths and weaknesses. Several factors should be considered for their use in routine targeted insertion applications in maize. First the system should be highly efficient to enable low cost production. It should support integration of large DNA molecules for multiple traits. Much of the studies described to date used small DNA molecules containing either the *PAT* or *ALS* selectable marker gene. The system should produce a high percentage of intact targeted insertion events without additional transformation vector inserts. The system should work in elite maize genetic backgrounds. The inserted trait genes function as expected. Finally, new trait gene inserts should not impact performance of the adjacent pre-existing trait genes.

### Site-directed integration mediated by site-specific recombinases

Another method of targeted integration relies on site-specific recombination systems, which mediate DNA exchange using sequence flanking the cross-over region (Sadowski, 1986). Site-specific recombination systems produce no DNA breaks during the process and yield a precise recombination product. The often studied recombinases that can mediate both intermolecular and intramolecular recombination include Lambda phage integrase (Int), Cre from the P1 phage and FLP from the yeast 2 μm plasmid.

Some site-specific recombination systems use a single recombinase protein to drive recombination between two identical or non-identical sites (Sadowski, 1986). Cre-*lox*, FLP-*FTR* and R-*RS* recombination systems have been widely explored for either sequence removal or targeted insertion in plants (Wang et al., 2011). For example, the Cre-*lox* system only requires only the Cre recombinase protein to mediate sequence exchange between two identical *lox* sites. Use of this type of site-specific recombination systems is much simpler than manipulating the endogenous plant recombination machinery. However, the reactions mediated by these recombinases are reversible. Compared with DNA excision, the DNA integration reaction is thermodynamically unfavorable. This is addressed by using mutant recognition sites that produce a less active recombination product (Albert et al., 1995; Day et al., 2000). Cre-mediated targeted integration has been demonstrated in tobacco, rice and soybean (Albert et al., 1995; Srivastava et al., 2004; Li et al., 2009, 2010). Based on marker gene expression, the FLP-*FRT* system produced targeted integration in maize, but the reports lack detailed molecular characterization data (Baszczynski et al., 2002; Lyznik et al., 2003).

The reversibility issue can also be addressed using another type of recombinases, i.e., integrases that mediate unidirectional recombination reactions. The reverse reactions mediated by integrases usually require additional proteins. The ΦC31 integrase mediates an irreversible reaction between two non-identical *att*B and *att*P sites (Belteki et al., 2003). Lambda integrase also requires two host factors, IHF*α* and IHFβ, to drive the reaction between two non-identical *att*B and *att*P sites, which generates two new, non-identical sites, *att*L, and *att*R. The reverse reaction between attL and attR requires an additional accessory factor XIS, in addition to IHF*α* and IHFβ, to regenerate *att*B and *att*P. These integrases have been used to mediate targeted insertion into plant genomes (Suttie et al., 2008; De Paepe et al., 2013).

Several groups were working to improve recombinase activity, incorporate heterospecific recognition sites and employ different vector designs that combine the various recombinases and recognition sites (Wang et al., 2011). Several reports described recombinase-mediated cassette exchange (RMCE) strategies that used different or mutated recombination sites and these studies had successfully mediated targeted insertion in plants (Baszczynski et al., 2002; Lyznik et al., 2003; Li et al., 2009).

## Perspectives

In contrast to maize nuclear genome transformation, there is no report of successful organelle genome transformation. The TALEN and CRISPR-Cas9 genome engineering tools may make these genomes accessible to modification. Epigenome information may identify more suitable loci for trait gene insertion. Regions similar to intergenic genomic safe harbor loci in the human genome (Sadelain et al., 2012) might also offer considerable benefit for transgene expression in plants. This may reduce the uncertainty associated random transgene insertion and improve the production of transgenic events to reduce product development cost.

It's also worth considering the benefits of integrating maize transformation with doubled haploid technology to improve the efficiency of the trait characterization process. It would be nice to produce plants homozygous for the trait during event generation. Aulinger et al. (2003) used gametic haploid embryos produced from another culture for biolistic transformation. After transformation haploid embryos were treated with a chromosome doubling agent like colchicine to produce fertile doubled haploid events. But they were unsuccessful, perhaps because maize anthers typically don't respond to another culture. An alternative might be to transform traditional maize haploid embryos derived from crosses of transformation host maize plants with a Stock6-derived haploid-inducer line (Geiger and Gordillo, 2009). This system would provide significant advantages over another culture, including genotype independence and more reliable and robust plantlet regeneration.

### Conflict of interest statement

The authors are employees of Syngenta which is actively involved in the research and development of technologies and products covered in this review. Several patent applications have been filed and some are pending in the area of plant transformation technologies.

## References

[B1] Adams W. Davis B. Kucher L. Lowe B. Spencer M. Mann M. T. (2005). Method and Apparatus for Substantially Isolating Plant Tissues. United States Patent Application Publication US2005/0246786 A1.

[B2] Ahmadabadi M. Ruf S. Bock R. (2007). A leaf-based regeneration and transformation system for maize (*Zea mays* L.). Transgenic Res. 16, 437–448 10.1007/s11248-006-9046-y 17103238

[B3] Ainley W. M. Sastry-Dent L. Welter M. E. Murray M. G. Zeitler B. Amora R. (2013). Trait stacking via targeted genome editing. Plant Biotechnol. J. 11, 1126–1134 10.1111/pbi.12107 23953646

[B4] Al-Abed D. Rudrabhatla S. Talla R. Goldman S. (2006). Split-seed: a new tool for maize researchers. Planta 223, 1355–1360 10.1007/s00425-006-0237-9 16489455

[B5] Albert H. Dale E. C. Lee E. Ow D. W. (1995). Site-specific integration of DNA into wild type and mutant lox sites placed in the plant genome. Plant J. 7, 649–659 10.1046/j.1365-313X.1995.7040649.x 7742860

[B6] Ananiev E. V. Wu C. Chamberlin M. A. Svitashev S. Schwartz C. Gordon-Kamm W. (2009). Artificial chromosome formation in maize (*Zea mays* L.). Chromosoma 118, 157–177 10.1007/s00412-008-0191-3 19015867

[B7] Armstrong C. L. Green C. E. (1985). Establishment and maintenance of friable, embryogenic maize callus and the involvement of L-proline. Planta 164, 207–214 24249562 10.1007/BF00396083

[B8] Armstrong C. L. Green C. E. Phillips R. L. (1991). Development and availability of germplasm with high Type II culture formation response. Maize Gen. Coop. Newsl. 65, 92–93

[B9] Aulinger I. E. Peter S. O. Schmid J. E. Stamp P. (2003). Gametic embryos of maize as a target for biolistic transformation: comparison to immature zygotic embryos. Plant Cell Rep. 21, 585–591 10.1007/s00299-002-0556-7 12789434

[B10] Ayer A. Wehrkamp-Richter S. Laffaire J.-B. Le Goff S. Levy J. Chaignon S. (2013). Gene targeting in maize by somatic ectopic recombination. Plant Biotechnol. J. 11, 305–314 10.1111/pbi.12014 23094946

[B11] Barreiro R. Cope J. M. Goldman D. M. Hunter J. L. Pellegrineschi A. (2011). Apparatus and Methods to Gain Access to and Extract Intact Immature Embryo from Developing Maize Kernels or Specific Internal Tissue or Structure from one or more Seeds. United States Patent Application Publication US2011/0054969 A1.

[B12] Baszczynski C. L. Bowen B. A. Peterson D. J. Tagliani L. A. (2002). Compositions and Methods to Stack Multiple Nucleotide Sequences of Interest in the Genome of a Plant. United States Patent Publication 6,455,315 B1.

[B13] Bayley C. Trolinder N. Ray C. Morgan M. Quisenberry J. E. Ow D. W. (1992). Engineering 2,4-D resistance in cotton. Theor. Appl. Genet. 83, 645–649 10.1007/BF00226910 24202683

[B14] Belhaj K. Chaparro-Garcia A. Kamoun S. Nekraso V. (2013). Plant genome editing made easy: targeted mutagenesis in model and crop plants using the CRISPR/Cas system. Plant Methods 9:39 10.1186/1746-4811-9-39 24112467 PMC3852272

[B15] Belteki G. Marina Gertsenstein M. Ow D. W. Nagy A. (2003). Site-specific cassette exchange and germline transmission with mouse ES cells expressing ϕC31 integrase. Nat. Biotechnol. 21, 321–324 10.1038/nbt787 12563279

[B16] Brettschneider R. Becker D. Lörz H. (1997). Efficient transformation of scutellar tissue of immature maize embryos. Theor. Appl. Genet. 94, 737–748 10.1007/s001220050473

[B17] Bullock W. P. (2011). Method and Apparatus for Extraction of Plant Embryos. United States Patent Application Publication US2011/0078819 A1.

[B18] Carlson S. R. Rudgers G. W. Zieler H. Mach J. M. Luo S. Grunden E. (2007). Meiotic transmission of an *in vitro*- assembled autonomous maize minichromosome. PLoS Genet. 3:e179 10.1371/journal.pgen.0030179 17953486 PMC2041994

[B19] Castle L. A. Siehl D. L. Gorton R. Patten P. A. Chen Y. H. Bertain S. (2004). Discovery and directed evolution of a glyphosate tolerance gene. Science 304, 1151–1154 10.1126/science.1096770 15155947

[B20] Chen Z. Zhong H. Kim M. Chilton M.-D. Richbourg L. Jiang Y. (2014). “TALEN- mediated targeted insertion of transgenes in maize. Abstract of FASEB Conference on “Genome Engineering: Cutting edge research and application”.

[B21] Cheng M. Lowe B. A. Spencer T. M. Ye X. Armstrong C. L. (2004). Factors influencing *Agrobacterium*-mediated transformation of monocotyledonous species. In Vitro Cell. Dev. Biol. Plant. 40, 31–45 10.1079/IVP2003501

[B22] D'Halluin K. Bonne E. Bossut M. De Beuckeleer M. Leemans J. (1992). Transgenic maize plants by tissue electroporation. Plant Cell 4, 1495–1505 1334743 10.1105/tpc.4.12.1495 PMC160236

[B23] D'Halluin K. Vanderstraeten C. Stals E. Cornelissen M. Ruiter R. (2008). Homologous recombination: a basis for targeted genome optimization in crop species such as maize. Plant Biotechnol. J. 6, 93–102 10.1111/j.1467-7652.2007.00305.x 17999657

[B24] D'Halluin K. Vanderstraeten C. Van Hulle J. Joanna Rosolowska J. Van Den Brande I. Pennewaert A. (2013). Targeted molecular trait stacking in cotton through targeted double-strand break induction. Plant Biotechnol. J. 11, 933–941 10.1111/pbi.12085 23777410 PMC4272417

[B25] Davis B. J. Mann M. T. (2006). Method for Excision of Plant Embryos for Transformation. United States Patent Publication 7,150,993.

[B26] Day C. D. Lee E. Kobayashi J. Holappa L. D. Albert H. Ow D. W. (2000). Transgene integration into the same chromosome location can produce alleles that express at a predictable level, or alleles that are differentially silenced. Genes Dev. 14, 2869–2880 10.1101/gad.849600 11090134 PMC317066

[B27] De Buck S. De Paepe A. Depicker A. (2013). Transgene expression in plants, Control of, in Sustainable Food Production, *ed P. Christou* (Springer), 1570–1593 10.1007/978-1-4614-5797-8_412

[B28] De Framond A. J. Black E. W. Chilton W. S. Kayes L. Chilton M.-D. (1986). Two unlinked T-DNAs can transform the same tobacco plant cell and segregate in the F1 generation. Mol. Gen. Genet. 202, 125–131 10.1007/BF00330528

[B29] De Paepe A. De Buck S. Nolf J. Van Lerberge E. Depicker A. (2013). Site-specific T–DNA integration in *Arabidopsis thaliana* mediated by the combined action of CRE recombinase and ΦC31 integrase. Plant J. 75, 172–184 10.1111/tpj.12202 23574114

[B30] Depicker A. Herman L. Jacobs A. Schell J. Van Montagu M. (1985). Frequencies of simultaneous transformation with different T-DNAs and their relevance to the *Agrobacterium*-plant cell interaction. Mol. Gen. Genet. 201, 477–484 10.1007/BF00331342

[B31] Djukanovic V. Smith J. Lowe K. Yang M. Gao H. Jones S. (2013). Male-sterile maize plants produced by targeted mutagenesis of the cytochrome P450-like gene (MS26) using a re-designed I-CreI homing endonuclease. Plant J. 76, 888–899 10.1111/tpj.12335 24112765

[B32] Duncan D. R. Williams M. E. Zehr B. E. Widholm J. M. (1985). The production of callus capable of plant regeneration from immature embryos of numerous *Zea mays* genotypes. Planta 165, 322–332 10.1007/BF00392228 24241136

[B33] Eby J. Held B. Hou L. Wilson H. (2004). Methods and Compositions for the Introduction of Molecules into Cells. United States Patent Application Publication US2004/0219676.

[B34] El-Itriby H. A. Assem S. K. Hussein E. H. A. Abdel-Galil F. M. Madkour M. A. (2003). Regeneration and transformation of Egyptian maize inbred lines via immature embryo culture and a biolistic particle delivery system. In Vitro Cell. Dev. Biol. Plant. 39, 524–531 10.1079/IVP2003439

[B35] Elkonin L. Pakhomova N. (2000). Influence of nitrogen and phosphorus on induction embryogenic callus of sorghum. Plant Cell Tissue Organ Cult. 61, 115–123 10.1023/A:1006472418218

[B36] Erikson O. Hertzberg M. Nasholm T. (2004). A conditional marker gene allowing both positive and negative selection in plants. Nat. Biotechnol. 22, 455–458 10.1038/nbt946 15085802

[B37] Frame B. R. Drayton P. R. Bagnall S. V. Lewnau C. J. Bullock W. P. Wilson H. M. (1994). Production of fertile transgenic maize plants by silicon carbide whisker-mediated transformation. Plant J. 6, 941–948 10.1046/j.1365-313X.1994.6060941.x

[B38] Frame B. R. McMurray J. M. Fonger T. M. Main M. L. Taylor K. W. Torney F. J. (2006). Improved *Agrobacterium*-mediated transformation of three maize inbred lines using MS salts. Plant Cell Rep. 25, 1024–1034 10.1007/s00299-006-0145-2 16710703

[B39] Frame B. R. Shou H. Chikwamba R. K. Zhang Z. Xiang C. Fonger T. M. (2002). *Agrobacterium tumefaciens*-mediated transformation of maize embryos using a standard binary vector system. Plant Physiol. 129, 13–22 10.1104/pp.000653 12011333 PMC1540222

[B40] Frame B. R. Zhang H. Cocciolone S. M. Sidorenko L. V. Dietrich C. R. Pegg S. E. (2000). Production of transgenic maize from bombarded type II callus: effect of particle size and callus morphology on transformation efficiency. In Vitro Cell. Dev. Biol. - Plant. 36, 21–29 10.1007/s11627-000-0007-5

[B41] Frary A. Hamilton C. M. (2001). Efficiency and stability of high molecular weight DNA transformation: an analysis in tomato. Transgenic Res. 10, 121–132 10.1023/A:1008924726270 11305359

[B42] Fromm M. E. Taylor L. P. Walbot V. (1986). Stable transformation of maize after gene transfer by electroporation. Nature 319, 791–793 10.1038/319791a0 3005872

[B43] Fromm M. Morrish F. Armstrong C. Williams R. Thomas J. Klein T. (1990). Inheritance and expression of chimeric genes in the progeny of transgenic maize plants. BioTechnology (N Y) 8, 833–839 10.1038/nbt0990-833 1366794

[B44] Fu X. D. Duc L. T. Fontana S. Bong B. B. Tinjuangjun P. Sudhakar D. (2000). Linear transgene constructs lacking vector backbone sequences generate low-copy-number transgenic plants with simple integration patterns. Transgenic Res. 9, 11–19 10.1023/A:1008993730505 10853265

[B45] Gao H. Smith J. Yang M. Jones S. Djukanovic V. Nicholson M. G. (2010). Heritable targeted mutagenesis in maize using a designed endonuclease. Plant J. 61, 176–187 10.1111/j.1365-313X.2009.04041.x 19811621

[B46] Geiger H. H. Gordillo G. A. (2009). Doubled haploids in hybrid maize breeding. Maydica 54, 485–499

[B47] Gelvin S. B. (2010). Plant proteins involved in *Agrobacterium*-mediated genetic transformation. Annu. Rev. Phytopathol. 48, 45–68 10.1146/annurev-phyto-080508-081852 20337518

[B57] Goodarzi H. Najafabadi H. S. Oikonomou P. Greco T. M. Fish L. Salavati R. (2012). Systematic discovery of structural elements governing stability of mammalian messenger RNAs. Nature 485, 264–268 10.1038/nature11013 22495308 PMC3350620

[B48] Gordon-Kamm W. Dilkes B. P. Lowe K. Hoerster G. Sun X. Ross M. (2002). Stimulation of the cell cycle and maize transformation by disruption of the plant retinoblastoma pathway. Proc. Natl. Acad. Sci. U.S.A. 99, 11975–11980 10.1073/pnas.142409899 12185243 PMC129379

[B49] Gordon-Kamm W. J. Spencer T. M. Mangano M. L. Adams T. R. Daines R. J. Start W. G. (1990). Transformation of maize cell and regeneration of fertile transgenic plants. Plant Cell 2, 603–618 10.1105/tpc.2.7.603 12354967 PMC159915

[B50] Gould J. Devey M. Hasegawa O. Ulian E. C. Peterson G. Smith R. H. (1991). Transformation of *Zea mays* L. using *Agrobacterium tumefaciens* and the shoot apex. Plant Physiol. 95, 426–434 10.1104/pp.95.2.426 16668001 PMC1077548

[B51] Graves A. C. F. Goldman S. L. (1986). The transformation of *Zea mays* seedlings with *Agrobacterium tumefaciens*. Plant Mol. Biol. 7, 43–50 10.1007/BF00020130 24302156

[B52] Green C. E. Philips R. L. (1975). Plant regeneration from tissue culture of maize. Crop Sci. 15, 417–421 10.2135/cropsci1975.0011183X001500030040x

[B53] Grimsley N. Hohn T. Davies J. W. Hohn B. (1987). *Agrobacterium*-mediated delivery of infectious maize streak virus into maize plants. Nature 325, 177–179 10.1038/325177a024272715

[B54] Gudynaite-Savitch L. Johnson D. A. Miki B. L. (2009). Strategies to mitigate transgene-promoter interactions. Plant Biotechnol J. 7, 472–485 10.1111/j.1467-7652.2009.00416.x 19490507

[B55] Guo X. M. Zhang X. D. Liang R. Q. Zhang L. Q. Chen Y. F. (2007). Maize transformation using xylose isomerase gene as a selection marker. J. Plant Physiol. Mol. Biol. 33, 547–552 18349509

[B56] Hamilton C. M. Frary A. Lewis C. Tanksley S. D. (1996). Stable transfer of intact high molecular weight DNA into plant chromosomes. Proc. Natl. Acad. Sci. U.S.A. 93, 9975–9979 10.1073/pnas.93.18.9975 8790442 PMC38540

[B58] Hansen G. (2000). Evidence for *Agrobacterium*-induced apoptosis in maize cells. Mol. Plant Microbe Interact. 13, 649–657 10.1094/MPMI.2000.13.6.649 10830264

[B59] Hansen G. Chilton M. D. (1996). Agrolistic” transformation of plant cells: integration of T-strands generated in planta. Proc. Natl. Acad. Sci. U.S.A. 93, 14978–14983 10.1073/pnas.93.25.14978 8962167 PMC26248

[B60] Hansen G. Wright M. S. (1999). Recent advances in the transformation of plants. Trends Plant Sci. 4, 226–231 10.1016/S1360-1385(99)01412-0 10366879

[B61] Hanson B. Engler D. York Moy Y. Newman B. Ralston E. Gutterson N. (1999). A simple method to enrich an *Agrobacterium*-transformed population for plants containing only T-DNA sequences. Plant J. 19, 727–734 10.1046/j.1365-313x.1999.00564.x 10571858

[B62] Harper B. McClain S. Ganko E. W. (2012). Interpreting the biological relevance of bioinformatic analyses with T-DNA sequence for protein allergenicity. Regul. Toxicol. Pharmacol. 63, 426–432 10.1016/j.yrtph.2012.05.014 22668749

[B63] Heck G. R. Armstrong C. L. Astwood J. D. Behr C. F. Bookout J. T. Brown S. M. (2005). Development and characterization of a CP4 EPSPS-based, glyphosate-tolerant corn event. Crop Sci. 44, 329–339 10.2135/cropsci2005.0329

[B64] Hellens R. Mullineaux P. Klee H. (2000). Technical focus: a guide to *Agrobacterium* binary Ti vectors. Trends Plant Sci. 5, 446–451 10.1016/S1360-1385(00)01740-4 11044722

[B65] Hiei Y. Ishida Y. Kasaoka K. Komari T. (2006). Improved frequency of transformation in rice and maize by treatment of immature embryos with centrifugation and heat prior to infection with *Agrobacterium tumefaciens*. Plant Cell Tissue Organ Cult. 87, 233–243 10.1007/s11240-006-9157-4

[B66] Hily J. M. Singer S. D. Yang Y. Liu Z. (2009). A transformation booster sequence (TBS) from Petunia hybrida functions as an enhancer-blocking insulator in *Arabidopsis thaliana*. Plant Cell Rep. 28, 1095–1104 10.1007/s00299-009-0700-8 19373469

[B69] Howe A. R. Gasser C. S. Brown S. M. Padgette S. R. Hart J. Parker G. B. (2002). Glyphosate as aselection agent for the production of fertile transgenic maize (*Zea mays* L.) plants. Mol. Breed. 10, 153–164 10.1023/A:1020396708088

[B70] Huang S. Gilbertson L. A. Adams T. H. Malloy K. P. Reisenbigler E. K. Birr D. H. (2004). Generation of marker free transgenics maize by regular two border *Agrobacterium transformation* vectors. Transgenic Res. 13, 451–461 10.1007/s11248-004-1453-3 15587269

[B71] Huang X. Wei Z. (2005). Successful Agrobacterium-mediated genetic transformation of maize elite inbred lines. Plant Cell Tissue Organ Cult. 83, 187–200 10.1007/s11240-005-5772-8

[B72] Ishida Y. Hiei Y. Komari T. (2007). *Agrobacterium*- mediated transformation of maize. Nat. Protoc. 2, 1614–1621 10.1038/nprot.2007.241 17585302

[B73] Ishida Y. Saito H. Ohta S. Hiei Y. Komari T. Kumashiro T. (1996). High efficiency transformation of maize (*Zea mays* L.) mediated by *Agrobacterium tumefaciens*. Nat. Biotechnol. 14, 745–750 10.1038/nbt0696-745 9630983

[B74] Jayne S. Barbour E. Meyer T. (2000). Methods for Improving Transformation Efficiency. United States Patent Publication 6,096,947.

[B75] Jones T. L. (2009). Maize tissue culture and transformation: the first 20 years, in Molecular Genetic Approaches to Maize Improvement, eds Kriz A. L. Larkins B. A. (Berlin; Heidelberg: Springer-Verlag), 6–26

[B76] Joshi C. P. Zhou H. Huang X. Chiang V. L. (1997). Context sequences of translation initiation codon in plants. Plant Mol. Biol. 35, 993–1001 10.1023/A:1005816823636 9426620

[B77] Kant P. Gulati A. Harris L. Gleddie S. Singh J. Pauls K. P. (2012). Transgenic corn plants with modified ribosomal protein L3 show decreased ear rot disease after inoculation with Fusarium graminearum. Austral. J. Crop Sci. 6, 1598–1605

[B78] Komari T. Hiei Y. Saito Y. Murai N. Kumashiro T. (1996). Vectors carrying two separate T-DNAs for co-transformation of higher plants mediated by *Agrobacterium tumefaciens* and segregation of transformants free from selection markers. Plant J. 10, 165–174 10.1046/j.1365-313X.1996.10010165.x 8758986

[B79] Kondrak M. van der Meer I. M. Banfalvi Z. (2006). Generation of marker- and backbone-free transgenic potatoes by site-specific recombination and a bi-functional marker gene in a non-regular one-border agrobacterium transformation vector. Transgenic Res. 15, 729–737 10.1007/s11248-006-9021-7 17072563

[B80] Kononov M. E. Bassuner B. Gelvin S. B. (1997). Integration of T-DNA binary vector “backbone” sequences into the tobacco genome: evidence for multiple complex patterns of integration. Plant J. 11, 945–957 10.1046/j.1365-313X.1997.11050945.x 9193068

[B81] Koziel M. G. Beland G. L. Bowman C. Carozzi N. B. Crenshaw R. Crossland L. (1993). Field performance of elite transgenic maize plants expressing an insecticidal protein derived from *Bacillus thuringiensis*. Biotechnology 11, 194–200 10.1038/nbt0293-194

[B82] Kuraya Y. Ohta S. Fukuda M. Hiei Y. Murai N. Hamada K. (2004). Suppression of transfer of non-T-DNA “vector backbone” sequences by multiple left border repeats in vectors for transformation of higher plants mediated by *Agrobacterium tumefaciens*. Mol. Breed. 14, 309–320 10.1023/B:MOLB.0000047792.77219.bb

[B83] Ladics G. S. Bannon G. A. Silvanovich A. Robert F. Cressman R. F. (2007). Comparison of conventional FASTA identity searches with the 80 amino acid sliding window FASTA search for the elucidation of potential identities to known allergens. Mol. Nutr. Food Res. 51, 985–998 10.1002/mnfr.200600231 17639511

[B84] Lai F. M. Mankin L. Mei K. F. Jones T. Song H. S. (2007). Selection System for Maize Transformation. World Intellectual Property Organization Publication WO 2007/014844 A2.

[B85] Lai F.-M. Privalle L. Mei K. Ghoshal D. Shen Y. Klucinec J. (2011). Evaluation of the *E. coli* D-serine ammonia lyase gene (*Ec. dsdA*) for use as a selectable marker in maize transformation. In Vitro Cell.Dev.Biol.- Plant. 47, 467–479 10.1007/s11627-011-9351-x

[B86] Le D. T. Choi J.-D. Tran L.-S. P. (2010). Amino acids conferring herbicide resistance in tobacco acetohydroxyacid synthase. GM Crops 1, 62–67 10.4161/gmcr.1.2.10856 21865873

[B87] Lee H. J. Duke M. V. Duke S. O. (1993). Cellular localization of protoporphyrinogen-oxidizing activities of etiolated barley (*Hordeum vulgare* L.) leaves (Relationship to mechanism of action of protoporphyrinogen oxidase-inhibiting herbicides). Plant Physiol. 102, 881–889 12231874 10.1104/pp.102.3.881 PMC158860

[B88] Lee L.-Y. Gelvin S. B. (2008). T-DNA Binary Vectors and Systems. Plant Physiol. 146, 325–332 10.1104/pp.107.113001 18250230 PMC2245830

[B89] Li G. Zhang Q. Zhang J. Bi Y. Shan L. (2002). Establishment of multiple shoot clumps from maize (*Zea mays* L.) and regeneration of herbicide-resistant transgenic plantlets. Sci. China C Life Sci. 45, 40–49 10.1360/02yc9005 18763062

[B90] Li X. Nicholl D. (2005). Development of PPO inhibitor-resistant cultures and crops. Pest Manag. Sci. 61, 277–285 10.1002/ps.1011 15660355

[B91] Li X. Sandy L. Volrath S. L. Nicholl D. B. G. Chilcott C. E. Johnson M. A. (2003). Development of protoporphyrinogen oxidase as an efficient selection marker for *Agrobacterium tumefaciens*-mediated transformation of maize. Plant Physiol. 133, 736–747 10.1104/pp.103.026245 12972658 PMC219048

[B92] Li Z. Moon B. P. Xing A. Liu Z.-B. McCardell R. P. Damude H. G. (2010). Stacking multiple transgenes at a selected genomic site via repeated recombinase-mediated DNA cassette exchanges. Plant Physiol. 154, 622–631 10.1104/pp.110.160093 20720171 PMC2949048

[B93] Li Z. Xing A. Moon B. P. McCardell R. P. Mills K. Falco S. C. (2009). Site-specific integration of transgenes in soybean via recombinase-mediated DNA cassette exchange. Plant Physiol. 151, 1087–1095 10.1104/pp.109.137612 19429604 PMC2773068

[B94] Liang Z. Zhang K. Chen K. Gao C. (2014). Targeted mutagenesis in *Zea mays* using TALENs and the CRISPR/Cas system. J. Genet. Genomics 41, 63–68 10.1016/j.jgg.2013.12.001 24576457

[B95] Liu Y.-G. Shirano Y. Fukaki H. Yanai Y. Tasaka M. Tabata S. (1999). Complementation of plant mutants with large genomic DNA fragments by a transformation-competent artificial chromosome vector accelerates positional cloning. Proc. Natl. Acad. Sci. U.S.A. 96, 6535–6540 10.1073/pnas.96.11.6535 10339623 PMC26917

[B96] Lowe K. Bowen B. Hoerster G. Ross M. Bond D. Pierce D. (1995). Germline transformation of maize following manipulation of chimeric shoot meristems. Biotechnology 13, 677–682 10.1038/nbt0795-677

[B97] Lowe K. Prakash N. S. Way M. Mann M. T. Spencer T. M. Boddupalli R. S. (2009). Enhanced single copy integration events in corn via particle bombardment using low quantities of DNA. Transgenic Res. 18, 831–840 10.1007/s11248-009-9265-0 19381853

[B98] Luerhsen K. Walbot V. (1994). The impact of AUG start codon context on maize gene expression *in vivo*. Plant Cell Rep. 13, 454–458 10.1007/BF00231966 24194025

[B99] Lyznik L. A. Gordon-Kamm W. J. Tao Y. (2003). Site-specific recombination for genetic engineering in plants. Plant Cell Rep. 21, 925–932 10.1007/s00299-003-0616-7 12835900

[B100] Lyznik L. A. Ryan R. D. Ritchie S. W. Hodges T. K. (1989). Stable cotransformation of maize protoplasts with gusA and neo genes. Plant Mol. Biol. 13, 151–161 10.1007/BF00016134 2562394

[B101] Martineau B. Voelker T. A. Sanders R. A. (1994). On defining T-DNA. Plant Cel. 6, 1032–1033 10.1105/tpc.6.8.1032 12244264 PMC160498

[B102] May B. P. Liu H. Vollbrecht E. Senior L. Rabinowicz P. D. Roh D. (2003). Maize-targeted mutagenesis: a knockout resource for maize. Proc. Natl. Acad. Sci. U.S.A. 100, 11541–11546 10.1073/pnas.1831119100 12954979 PMC208794

[B103] McCutchen B. F. Hazel C. B. Liu D. L. Lu A. L. Wayne J. M. Olson P. D. (2007). Methods and Compositions for the Expression of a Polynucleotide of Interest. United States Patent Application Publication US2007/0061917 A1.

[B104] McKnight T. D. Lillis M. T. Simpson R. B. (1987). Segregation of genes transferred to one plant cell from two separate *Agrobacterium* strains. Plant Mol. Biol. 8, 439–445 10.1007/BF00017989 24301306

[B105] Mets L. J. (1993). Aerosol Beam Microinjector. United States Patent Number 5,240,842

[B106] Meyer P. Saedler H. (1996). Homology-dependent gene silencing in plants. Annu. Rev. Plant Physiol. Plant Mol. Biol. 47, 23–48 10.1146/annurev.arplant.47.1.23 15012281

[B107] Miller M. Tagliani L. Wang N. Berka B. Bidney D. Zhao Z. Y. (2002). High efficiency transgene segregation in co-transformed maize plants using an *Agrobacterium tumefaciens* 2 T-DNA binary system. Transgenic Res. 11, 381–396 10.1023/A:1016390621482 12212841

[B108] Negrotto D. Jolley M. Beer S. Wench A. R. Hansen G. (2000). The use of phosphomannose- isomerase as a selectable marker to recover transgenic maize plants (*Zea mays* L.) via *Agrobacterium* transformation. Plant Cell Rep. 19, 798–803 10.1007/s002999900187 30754872

[B109] O'Connor-Sánchez A. Cabrera-Ponce J. L. Valdez-Melara M. Téllez-Rodríguez P. Pons-Hernández J. L. Herrera-Estrella L. (2002). Transgenic maize plants of tropical and subtropical genotypes obtained from calluses containing organogenic and embryogenic-like structures derived from shoot tips. Plant Cell Rep. 21, 302–312 10.1007/s00299-002-0502-8

[B110] Oltmanns H. Frame B. Lee L.-Y. Johnson S. Li B. Wang K. (2010). Generation of backbone-free, low transgene copy plants by launching T-DNA from the *Agrobacterium* chromosome. Plant Physiol. 152, 1158–1166 10.1104/pp.109.148585 20023148 PMC2832237

[B111] Ombori O. Gitonga N. M. Machuka J. (2008). Somatic embryogenesis and plant regeneration from immature embryos of tropical maize (*Zea mays* L.) inbred lines. Biotechnology 7, 224–232 10.3923/biotech.2008.224.232

[B112] Ombori O. Muoma J. V. O. Machuka J. (2013). *Agrobacterium*-mediated genetic transformation of selected tropical inbred and hybrid maize (*Zea mays* L.) lines. Plant Cell Tissue Organ Cult. 113, 11–23 10.1007/s11240-012-0247-1

[B113] Omirulleh S. Abraham M. Golovkin M. Stefanov I. Karabaev M. K. Mustardy L. (1993). Activity of a chimeric promoter with the doubled CaMV 35S enhancer element in protoplast-derived cells and transgenic plants in maize. Plant Mol. Biol. 21, 415–428 10.1007/BF00028800 8443339

[B114] Park S. H. Lee B.-M. Salas M. G. Srivatanakul M. Smith R. H. (2000). Shorter T-DNA or additional virulence genes improve *Agrobacterium*-mediated transformation. Theor. Appl. Genet. 101, 1015–1020 10.1007/s00122005157510092170

[B115] Puchta H. Dujon B. Hohn B. (1996). Two different but related mechanisms are used in plants for the repair of genomic double-strand breaks by homologous recombination. Proc. Natl. Acad. Sci. U.S.A. 93, 5055–5060 10.1073/pnas.93.10.5055 8643528 PMC39405

[B116] Puchta H. Fauser F. (2014). Synthetic nucleases for genome engineering in plants: prospects for a bright future. Plant J. 78, 727–741 10.1111/tpj.12338 24112784

[B117] Que Q. (2006). Targeted Integration and Stacking of Dna Through Homologous Recombination. United States Patent Application Publication US2006/0253918 A1.

[B118] Que Q. Chilton M.-D. M. de Fontes C. M. He C. Nuccio M. Zhu T. (2010). Trait stacking in transgenic crops: challengs and opportunities. GM Crops 1, 220–229 10.4161/gmcr.1.4.13439 21844677

[B119] Que Q. Nicholl D. (2012). Enhanced Transformation of Recalcitrant Monocots. United States Patent Application Publication US2012/0278950 A1.

[B120] Register J. C. III. Peterson D. J. Bell P. J. Bullock W. P. Evans I. J. Frame B. (1994). Structure and function of selectable and non-selectable transgenes in maize after introduction by particle bombardment. Plant Mol. Biol. 25, 951–961 10.1007/BF00014669 7919215

[B121] Rhodes C. A. Lowe K. S. Ruby K. L. (1988b). Plant regeneration from protoplasts isolated from embryogenic maize cell cultures. Biotechnology 6, 56–60

[B122] Rhodes C. A. Pierce D. A. Mettler I. J. Mascarenhas D. Detmer J. J. (1988a). Genetically transformed maize plants from protoplasts. Science 240, 204–207 2832947 10.1126/science.2832947

[B123] Rout J. R. Lowe B. A. Purcell J. Spelletich A. Spencer M. Way M. (2013). Method of Rapidly Transforming Monocots. United States Patent Publication 8,395,020.

[B124] Sadelain M. Papapetrou E. P. Bushman F. D. (2012). Safe harbours for the integration of new DNA in the human genome. Nat. Rev. Cancer 12, 51–58 10.1038/nrc3179 22129804

[B125] Sadowski P. (1986). Site-specific recombinases: changing partners and doing the twist. J. Bacteriol. 165, 341–347 3003022 10.1128/jb.165.2.341-347.1986 PMC214421

[B126] Sairam R. V. Parani M. Franklin G. Lifeng Z. Smith B. MacDougall J. (2003). Shoot meristem: an ideal explant for *Zea mays* L. transformation. Genome 46, 323–329 10.1139/g02-120 12723048

[B127] Sheng J. Citovsky V. (1996). *Agrobacterium*-plant cell DNA transport: have virulence proteins, will travel. Plant Cell 8, 1699–1710 10.1105/tpc.8.10.1699 8914322 PMC161308

[B174] She W. Lin W. Zhu Y. Chen Y. Jin W. Yang Y. (2010). The *gypsy* insulator of *Drosophila melanogaster*, together with its binding protein suppressor of Hairy-Wing, facilitate high and precise expression of transgenes in *Arabidopsis thaliana*. Genetics 185, 1141–1150 10.1534/genetics.110.117960 20516496 PMC2922898

[B128] Shillito R. D. Carswell G. K. Johnson C. M. DiMaio J. J. Harms C. T. (1989). Regeneration of fertile plants from protoplasts of elite inbred maize. Biotechnology 7, 581–587 10.1038/nbt0689-581

[B129] Shukla V. K. Doyon Y. Miller J. C. DeKelver R. C. Moehle E. A. Worden S. E. (2009). Precise genome modification in the crop species *Zea mays* using zinc-finger nucleases. Nature 459, 437–441 10.1038/nature07992 19404259

[B130] Sidorov V. Gilbertson L. Addae P. Duncan D. (2006). *Agrobacterium*-mediatd transformation of seedling-derived maize callus. Plant Cell Rep. 25, 320–328 10.1007/s00299-005-0058-5 16252091

[B131] Silvanovich A. Bannon G. McClain S. (2009). The use of *E*-score to determine the quality of protein alignments. Regul. Toxicol. Pharmacol. 54, S26–S31 10.1016/j.yrtph.2009.02.004 19245824

[B132] Simoens C. Alliotte T. Mendel R. Muller A. Schiemann J. Van Lijsebettens M. (1986). A binary vector for transferring genomic libraries to plants. Nucleic Acids Res. 14, 8073–8090 10.1093/nar/14.20.8073 3534794 PMC311835

[B133] Singer S. D. Cox K. D. Liu Z. (2011). Enhancer-promoter interference and its prevention in transgenic plants. Plant Cell Rep. 30, 723–731 10.1007/s00299-010-0977-7 21170713

[B134] Sivamani E. DeLong R. K. Qu R. (2009). Protamine-mediated DNA coating remarkably improves bombardment transformation efficiency in plant cells. Plant Cell Rep. 28, 213–221 10.1007/s00299-008-0636-4 19015859

[B135] Songstad D. D. Armstrong C. L. Peterson W. L. (1991). AgNO3 increases type II callus production from immature embryos of maize inbred B73 and its derivatives. Plant Cell Rep. 9, 699–702 10.1007/BF00235361 24213697

[B136] Songstad D. D. Armstrong C. L. Peterson W. L. Hairston B. Hinchee M. A. W. (1996). Production of transgenic maize plants and progeny by bombardment of Hi-II immature embryos. In Vitro Cell Dev. Biol. Plant 32, 179–183 10.1007/BF02822763

[B137] Spencer T. M. Gordon-Kamm W. J. Daines R. J. Start W. G. Lemaux P. G. (1990). Bialaphos selection of stable transformants from maize cell culture. Theor. Appl. Genet. 79, 625–631 10.1007/BF00226875 24226576

[B138] Srivastava V. Ariza-Nieto M. Wilson A. J. (2004). Cre-mediated site-specific gene integration for consistent transgene expression in rice. Plant Biotechnol. J. 2, 169–179 10.1111/j.1467-7652.2003.00061.x 17147608

[B139] Srivastava V. Ow D. W. (2001). Single copy primary transformants of maize obtained through the co-introduction of a recombinase-expressing construct. Plant Mol. Biol. 46, 561–566 10.1023/A:1010646100261 11516149

[B140] Stuiver M. H. Ponstein A. S. Ohl S. A. Goddijn O. J. M. Simons L. H. Dekker B. M. M. (2000). Plasmids for Plant Transformation and Method for Using the Same. United States Patent Publication 7,029,908.

[B141] Sugita K. Kasahara T. Matsunaga E. Ebinuma H. (2000). A transformation vector for the production of marker-free transgenic plants containing a single copy transgene at high frequency. Plant J. 22, 461–469 10.1046/j.1365-313X.2000.00745.x 10849362

[B142] Sundar I. K. Sakthivel N. (2008). Advances in selectable marker genes for plant transformation. J. Plant Physiol. 165, 1698–1716 10.1016/j.jplph.2008.08.002 18789557

[B143] Suttie J. L. Chilton M. D. Que Q. (2008). Lambda Integrase Mediated Recombination in Plants. United States Patent Publication 7,351,877.

[B144] Swanda A. P. Starr R. A. Jamruszka-Lewis A. M. (2013). Automated System and Method for Separating and Sigulating Plant Embryos. United States Patent Application Publication US2013/0167438 A1.

[B145] Till B. J. Reynolds S. H. Weil C. Springer N. Burtner C. Young K. (2004). Discovery of induced point mutations in maize genes by TILLING. BMC Plant Biol. 4:12 10.1186/1471-2229-4-12 15282033 PMC512284

[B146] Timmis R. Hirahara E. Folster H. G. Surerus-Lopez H. (2004). Automated System and Method for Harvesting and Multi-Stage Screening of Plant Embryos. United States Patent Application Publication US2004/0267457 A1.

[B147] Tinland B. Schoumacher F. Gloeckler V. Bravo-Angel A. M. Hohn B. (1995). The *Agrobacterium tumefaciens* virulence D2 protein is responsible for precise integration of T-DNA into the plant genome. EMBO J. 14, 3585–3595 7628458 10.1002/j.1460-2075.1995.tb07364.x PMC394426

[B148] Tisserat B. Vandercook C. (1985). Development of an automated plant culture system. Plant Cell Tissue Organ Cult. 5, 107–117 10.1007/BF00040307

[B149] Vain P. McMullen M. D. Finer J. J. (1993). Osmotic treatment enhances particle bombardment- mediated transient and stable transformaiton of maize. Plant Cell Rep. 12, 84–88 10.1007/BF00241940 24202074

[B150] Valdez-Ortiz A. Merdina-Godoy S. Valverde M. E. Paredes-Lo'pez O. (2007). A transgenic tropical maize line generated by the direct transformation of the embryo- scutellum by *A. tumefaciens*. Plant Cell Tissue Organ Cult. 91, 201–214 10.1007/s11240-007-9286-4

[B151] Vega J. M. Yu W. Kennon A. R. Chen X. Zhang Z. J. (2008). Improvement of *Agrobacterium*-mediated transformation in Hi-II maize (*Zea mays*) using standard binary vectors. Plant Cell Rep. 27, 297–305 10.1007/s00299-007-0463-z 17938932

[B152] Voytas D. F. (2013). Plant genome engineering with sequence-specific nucleases. Annu. Rev. Plant Biol. 64, 327–350 10.1146/annurev-arplant-042811-105552 23451779

[B153] Walters D. Vetsch C. Potts D. Lundquist R. (1992). Transformation and inheritance of a hygromycin phosphotransferase gene in maize plants. Plant Mol. Biol. 18, 189–200 10.1007/BF00034948 1310057

[B154] Wan Y. Widholm J. M. Lemaux P. G. (1995). Type I callus as a bombardment target for generating fertile transgenic maize *(Zea mays L.)*. Planta 196, 7–14 10.1007/BF00193211

[B155] Wang A. S. Evans R. A. Altendorf P. R. Hanten J. A. Doyle M. C. Rosichan J. L. (2000). A mannose selection system for production of fertile transgenic maize plants from protoplasts. Plant Cell Rep. 19, 654–660 10.1007/s002999900181 30754801

[B156] Wang K. Frame B. Ishida Y. Komari T. (2009). Maize transformation, in Handbook of Maize: Genetics and Genomics, eds Bennetzen J. L. Hake S. (Springer Science + Business Media LLC), 609–639 10.1007/978-0-387-77863-1

[B157] Wang Y. Yau Y. Y. Perkins-Balding D. Thomson J. G. (2011). Recombinase technology: applications and possibilities. Plant Cell Rep. 30, 267–285 10.1007/s00299-010-0938-1 20972794 PMC3036822

[B158] Wright M. Dawson J. Dunder E. Suttie J. Reed J. Kramer C. (2001). Efficient biolistic transformation of maize (*Zea mays* L.) and wheat (*Triticum aestivum* L.) using the phosphomannose isomerase gene, *pmi*, as the selectable marker. Plant Cell Rep. 20, 429–436 10.1007/s002990100318 24549451

[B159] Wright T. R. Shan G. M. Walsh T. A. Lira J. M. Cui C. Song P. (2010). Robust crop resistance to broadleaf and grass herbicides provided by aryloxyalkanoate dioxygenase transgenes. Proc. Natl. Acad. Sci. U.S.A. 107, 20240–20245 10.1073/pnas.1013154107 21059954 PMC2996712

[B160] Xiayi K. Xiuwen Z. Heping S. Baojian L. (1996). Electroporation of immature maize zygotic embryos and regeneration of transgenic plants. Transgenic Res. 5, 219–221 10.1007/BF019697131334743

[B161] Ye X. Williams E. J. Shen J. Esser J. A. Nichols A. M. Petersen M. W. (2008). Plant development inhibitory genes in binary vector backbone improve quality event efficiency in soybean transformation. Transgenic Res. 17, 827–838 10.1007/s11248-008-9169-4 18253857

[B162] Ye X. Williams E. J. Shen J. Johnson S. Lowe B. Radke S. (2011). Enhanced production of single copy backbone-free transgenic plants in multiple crop species using binary vectors with a pRi replication origin in *Agrobacterium tumefaciens*. Transgenic Res. 20, 773–786 10.1007/s11248-010-9458-6 21042934

[B163] Yu G.-R. Liu Y. Du W.-P. Song J. Lin M. Xu L.-S. (2013). Optimization of *Agrobacterium tumefaciens*-mediated immature embryo transformation system and transformation of glyphosate-resistantg ene 2mG2-EPSPS in maize (*Zea mays* L.). J. of Integrative Agricult. 12, 2134–2142

[B164] Zhang S. Williams-Carrier R. Lemaux P. G. (2002). Transformation of recalcitrant maize elite inbreds using *in vitro* shoot meristematic cultures induced from germinated seedlings. Plant Cell Rep. 21, 263–270 10.1007/s00299-002-0513-5

[B165] Zhang W.-J. Dewey R. E. Boss W. Phillippy B. Q. Qu R. (2013). Enhanced *Agrobacterium*-mediated transformation efficiencies in monocot cells is associated with attenuated defense responses. Plant Mol. Biol. 81, 273–286 10.1007/s11103-012-9997-8 23242917

[B166] Zhang W. Subbarao S. Addae P. Shen A. Armstrong C. Peschke V. (2003). Cre/lox-mediated marker gene excision in transgenic maize (*Zea mays* L.) plants. Theor. Appl. Genet. 107, 1157–1168 10.1007/s00122-003-1368-z 14513214

[B167] Zhao C.-H. Zhang L.-J. Ge C. Hu K. (2008). Establishment and optimization of the regeneration system of mature embryos of maize (*Zea mays* L.). Agricult. Sci. China 7, 1046–1051 10.1016/S1671-2927(08)60145-5

[B168] Zhao Z-Y. Gu W. Cai T. Tagliani L. Hondred D. Bond D. (2001). High throughput genetic transformation mediated by *Agrobacterium tumefaciens* in maize. Mol. Breed. 8, 323–333 10.1023/A:1015243600325

[B169] Zhong H. Sun B. Warkentin D. Zhang S. Wu R. Wu T. (1996). The competence of maize shoot meristems for integrative transformation and inherited expression of transgenes. Plant Physiol. 110, 1097–1107 12226244 10.1104/pp.110.4.1097 PMC160889

[B170] Zhu T. Mettenburg K. Peterson D. J. Tagliani L. Baszczynski C. L. (2000). Engineering herbicide-resistant maize using chimeric RNA/DNA oligonucleotides. Nat. Biotechnol.18, 556–558 10.1038/75435 10802626

[B171] Zhu T. Peterson D. J. Tagliani L. St. Clari G. Baszczynski C. L. Bowen B. (1999). Targeted manipulation of maize genes *in vivo* using chimeric RNA/DNA oligonucleotides. Proc. Natl. Acad. Sci. U.S.A. 96, 8768–8773 10.1073/pnas.96.15.8768 10411950 PMC17591

[B172] Ziemienowicz A. Shim Y.-S. Matsuoka A. Eudes F. Kovalchuk I. (2012). A novel method of transgene delivery into *Triticale* plants using the *Agrobacterium* transferred DNA-derived nano-complex. Plant Physiol. 158, 1503–1513 10.1104/pp.111.192856 22291201 PMC3320166

[B173] Zuo J. Niu Q. W. Moller S. G. Chua N. H. (2001). Chemical-regulated, site-specific DNA excision in transgenic plants. Nat. Biotechnol. 19, 157–161 10.1038/84428 11175731

